# Disrupted properties of functional brain networks in major depressive disorder during emotional face recognition: an EEG study via graph theory analysis

**DOI:** 10.3389/fnhum.2024.1338765

**Published:** 2024-02-13

**Authors:** Chao-Lin Teng, Lin Cong, Wei Wang, Shan Cheng, Min Wu, Wei-Tao Dang, Min Jia, Jin Ma, Jin Xu, Wen-Dong Hu

**Affiliations:** ^1^Department of Aerospace Medicine, Air Force Medical University, Xi’an, Shaanxi, China; ^2^Department of Psychiatry, The First Affiliated Hospital of Xi’an Jiaotong University, Xi’an, Shaanxi, China; ^3^The Key Laboratory of Biomedical Information Engineering of Ministry of Education, Institute of Health and Rehabilitation Science, School of Life Science and Technology, Xi’an Jiaotong University, Xi’an, Shaanxi, China

**Keywords:** major depressive disorder (MDD), electroencephalography (EEG), emotional face recognition, functional brain network, phase transfer entropy (PTE)

## Abstract

Previous neuroimaging studies have revealed abnormal brain networks in patients with major depressive disorder (MDD) in emotional processing. While any cognitive task consists of a series of stages, little is yet known about the topology of functional brain networks in MDD for these stages during emotional face recognition. To address this problem, electroencephalography (EEG)-based functional brain networks of MDD patients at different stages of facial information processing were investigated in this study. First, EEG signals were collected from 16 patients with MDD and 18 age-, gender-, and education-matched normal subjects when performing an emotional face recognition task. Second, the global field power (GFP) method was employed to divide group-averaged event-related potentials into different stages. Third, using the phase transfer entropy (PTE) approach, the brain networks of MDD patients and normal individuals were constructed for each stage in negative and positive face processing, respectively. Finally, we compared the topological properties of brain networks of each stage between the two groups using graph theory approaches. The results showed that the analyzed three stages of emotional face processing corresponded to specific neurophysiological phases, namely, visual perception, face recognition, and emotional decision-making. It was also demonstrated that depressed patients showed abnormally decreased characteristic path length at the visual perception stage of negative face recognition and normalized characteristic path length in the stage of emotional decision-making during positive face processing compared to healthy subjects. Furthermore, while both the MDD and normal groups’ brain networks were found to exhibit small-world network characteristics, the brain network of patients with depression tended to be randomized. Moreover, for patients with MDD, the centro-parietal region may lose its status as a hub in the process of facial expression identification. Together, our findings suggested that altered emotional function in MDD patients might be associated with disruptions in the topological organization of functional brain networks during emotional face recognition, which further deepened our understanding of the emotion processing dysfunction underlying MDD.

## 1 Introduction

Major depressive disorder (MDD) is a kind of mental disorder with a persistently low state of mood (Malhi and Mann, 2018; Monroe and Harkness, 2022), which is a major contributor to abnormal behavior and cognition in relation to emotion. As an external expression of emotion, the emotional face plays an essential role in daily and social communication. Furthermore, recognizing facial expressions enables us to acquire lots of basic information about people, including gender, age, feelings, and attitudes, which further facilitates mutual understanding in social interactions (Lei et al., 2021; Liu et al., 2021). However, this important function in human life is dramatically disrupted by MDD, resulting in the dysfunction of emotional processing in depressed patients (Kaiser et al., 2015; Li and Wang, 2021; Slonim et al., 2023). Notably, over 350 million people were suffering from major depression, according to a report by the World Health Organization (Smith, 2014). In addition, it was reported that approximately 850,000 people with depression choose to take their own lives every year (Hu et al., 2023). Given this widespread influence, focusing priority investigations on emotion-related studies in individuals with MDD has a significant impact on the improvement of objective diagnosis and treatment of the disease in clinical practice.

By using an eye-tracking method, ample evidence has been repeatedly reported that MDD is associated with aberrant attentional bias during negative face recognition (Lazarov et al., 2018; Klawohn et al., 2020; Kuehl et al., 2021). For instance, Klawohn et al. (2020) found that the depressed group dwelled longer on sad faces than healthy people. A systematic review by Huang et al. (2023) also pointed out the negative attention bias toward emotional stimuli regarding eye-tracking. On the other hand, an increasing amount of studies based on functional magnetic resonance imaging (fMRI) have also revealed disrupted brain function of depression patients in response to emotional faces. For example, Hall et al. (2014) demonstrated that adolescents with MDD exhibited hypoactivation in the insula and temporal gyrus when viewing fearful faces. Moreover, a study by Henje Blom et al. (2015) pointed out that in comparison with normal subjects, MDD patients showed significantly decreased activation in the lingual and fusiform gyrus and increased activation in the thalamus for the contrast of happy versus sad faces. In addition, several fMRI connectivity studies using emotional face processing tasks showed extensive disruptions between different brain regions in depression, such as reduced functional connectivity between subgenual anterior cingulate (sgACC) and other regions (e.g., fusiform gyrus and precuneus) (Ho et al., 2014), increased functional connectivity of the insular cortex and fusiform/frontal/amygdala gyrus (Henje Blom et al., 2015), decreased connectivity from amygdala to sgACC, and increased fusiform face area-sgACC connectivity (Willinger et al., 2022). Taken together, these studies illustrated the dysfunction of facial expression identification underlying MDD involved in multiple brain regions rather than specific local alterations, which may play key roles in affective processing. However, the human brain responds to any cognitive process very quickly (the millisecond scale), and this rapidly changing neuronal activity is barely tracked by fMRI signals due to its slow nature (Brookes et al., 2014).

As an electrophysiological neuroimaging technique, event-related potential (ERP) obtained by the average of event-related electroencephalography (EEG) epochs directly reflects neuronal electrical activity with high temporal resolution, which can allow researchers to capture the dynamic changes of brain activity at a sub-second time scale. Until now, numerous research studies on emotional face processing in MDD have mainly focused on ERP components, such as P1, P2, N170, P3, and late positive potential (LPP) (Zhao et al., 2015; Zhang et al., 2016; Burkhouse et al., 2017; Ao et al., 2020; Tong et al., 2020; Chilver et al., 2022; Li et al., 2023), most of which consistently reported the abnormality of N170 in patients with MDD. Specifically, a number of studies showed the N170 component closely associated with the neural mechanisms of facial expression identification (Rossion, 2014; Damaskinou and Watling, 2018; Lai et al., 2020). These ERP studies only struggled with a single brain region, whereas a cognitive task depended on interactions between different regions in the entire large-scale functional brain network (Bressler and Menon, 2010). Hence, the brain network analysis provided a new insight into the dysfunction of MDD. An EEG study by Li et al. (2015) indicated that the depressed patients’ brain network tended to be a random network for the whole emotional face processing compared to healthy subjects. Liu et al. (2020) analyzed the EEG data of depression patients during music perception, and the results showed that compared to normal people, patients with MDD exhibited significantly decreased network connections within frontal brain areas and smaller clustering coefficient and characteristic path length in the beta frequency, indicating that the MDD group had a trend toward random networks. Actually, any cognitive process can be divided into several transiently stable stages within fast transitions, and each stage is characterized by a specific functional brain network (Hassan et al., 2015; Mheich et al., 2015). Therefore, exploring the spatiotemporal dynamics of networks during the face recognition process contributes to our further understanding of the brain mechanism of emotional dysfunction in patients with depression. However, to the best of our knowledge, few studies have yet investigated the topological organization of functional brain networks in MDD patients at different stages during emotional face recognition.

Since the human brain is one of the most complex systems in the world, the study can elucidate meaningful information regarding the brain network from the viewpoint of the direction of interactions between brain regions. In the present study, a well-known effective connectivity method, phase transfer entropy (PTE), proposed by Lobier et al. (2014), was applied to construct brain networks. As the extension of transfer entropy, the PTE method not only estimates the strength and direction of connection but also contains linear and non-linear couplings between neural signals by using phase data (Lobier et al., 2014). Furthermore, the PTE benefits from the phase information of analyzed signals, which demonstrated that phase patterns could reveal more comprehensive information than amplitude during visual and auditory processing and resting state (Schyns et al., 2011; Ng et al., 2013; von Wegner et al., 2021).

In this study, based on scalp EEG, functional brain networks of patients with MDD at different stages in the process of emotional face recognition were investigated. First, EEG signals were recorded from MDD patients and healthy controls during facial expression processing. Next, by using global field power (GFP), group-averaged ERP signals were segmented into successive stages for the depressed and normal groups. The PTE method was then adopted to establish brain networks for each stage for each group. Finally, we compared the properties of network topological organization between two groups through graph theory-based approaches.

## 2 Materials and methods

### 2.1 Participants

Thirty-four right-handed participants voluntarily took part in this study: 16 drug-naive, first-episode MDD patients in the MDD group recruited from the First Affiliated Hospital of Medical College of Xi’an Jiaotong University and 18 healthy controls (HCs) in the HC group without a family history of neurological and psychiatric diseases from Xi’an Jiaotong University. The diagnosis of MDD was primarily confirmed by expert psychiatrists based on the Diagnostic and Statistical Manual of Mental Disorders, Fifth Edition (DSM-5). MDD patients were also assessed using the 17-item Hamilton Depression Rating Scale (HAMD), and all had an HAMD score equal to or greater than 18. All patients had no organic brain disorders or other physical diseases. None of the patients have taken any antipsychotics and received any psychotherapy or physiotherapy (e.g., electroconvulsive therapy) for at least a month. To identify MDD patients with bipolar disorder, the most important point was to thoroughly collect information about the patient’s medical history from the patient and their families. This would allow the psychiatrists to know if the patient has taken antidepressant-related drugs in the past, if the depression was the first-episode, and if the patient has experienced manic symptoms such as high mood, high energy, and inflated ego over a period of time. Then, some mania scales, including the Bech-Rafaelsen Mania Rating Scale (BRMS) and the Young Mania Rating Scale (YMRS), were applied to further evaluate manic manifestations to exclude mania. Finally, depression was distinguished from bipolar disorder by combining the diagnostic criteria of mental diseases. Subjects in the HC group were carefully screened through a clinical interview and scored equal to or less than 7 on the HAMD. Moreover, healthy volunteers who were taking psychoactive drug treatment were excluded. The means and standard deviations (SD) of demographic information and clinical measures for the MDD and HC groups are provided in Table 1. Statistical results show no significant differences between the two groups for gender, age, education, and Mini-Mental State Examination (MMSE) scores (*p* > 0.05).

**Table 1:** Demographic information and clinical measures of healthy controls and patients with MDD (mean ± SD).

	HC group	MDD group	value of *p*
Male/Female	8/10	5/11	0.429^a^
Age (years)	24.94 ± 5.00	27.38 ± 9.52	0.825^b^
Education (years)	15.61 ± 2.73	15.13 ± 1.67	0.384^b^
HAMD scores	2.06 ± 2.11	23.88 ± 5.06	<0.001^b^
MMSE scores	29.61 ± 0.50	29.50 ± 0.73	0.878^b^

^a^ chi-square test; ^b^ Mann–Whitney *U*-test.

HAMD, Hamilton Depression Scale Rating; MMSE, Mini-Mental State Examination; HC, healthy control; MDD, major depressive disorder.

Additionally, both the MDD group and HC group reported no history of alcohol or substance abuse dependency. All enrolled participants had normal or corrected-to-normal visual acuity and no color blindness. The study procedures were approved by the ethics committee of the hospital, and each subject signed the written informed consent to participate in this study before the experiment began.

### 2.2 Stimulus materials

All facial stimulus materials used in the study were taken from the official website of Visual China. Each face picture selected by us was similar in spatial frequency, brightness, background, and contrast grade. We also applied Adobe Photoshop software to process all the pictures to make each picture with the same resolution (450 × 600px) and the same gray background. On the one hand, to make facial expressions easy to identify, crying, sad, and angry faces were selected as negative emotions, happy and cheerful faces as positive emotions, and expressionless faces as neutral emotions. On the other hand, we recruited 60 normal volunteers to rate each picture with a score range of −3 to 3 (−3: very negative; −2: negative; −1: little negative; 0: neutral; 1: little positive; 2: positive; 3: very positive) to validate whether they correctly evoked emotion in the test subjects or not. Ultimately, 68 pictures were rated a score of −3 by every volunteer, 68 pictures with 3 scores, and 128 pictures with 0 scores were picked out as negative, positive, and neutral faces, respectively.

### 2.3 Emotional face recognition task

**Figure 1:**
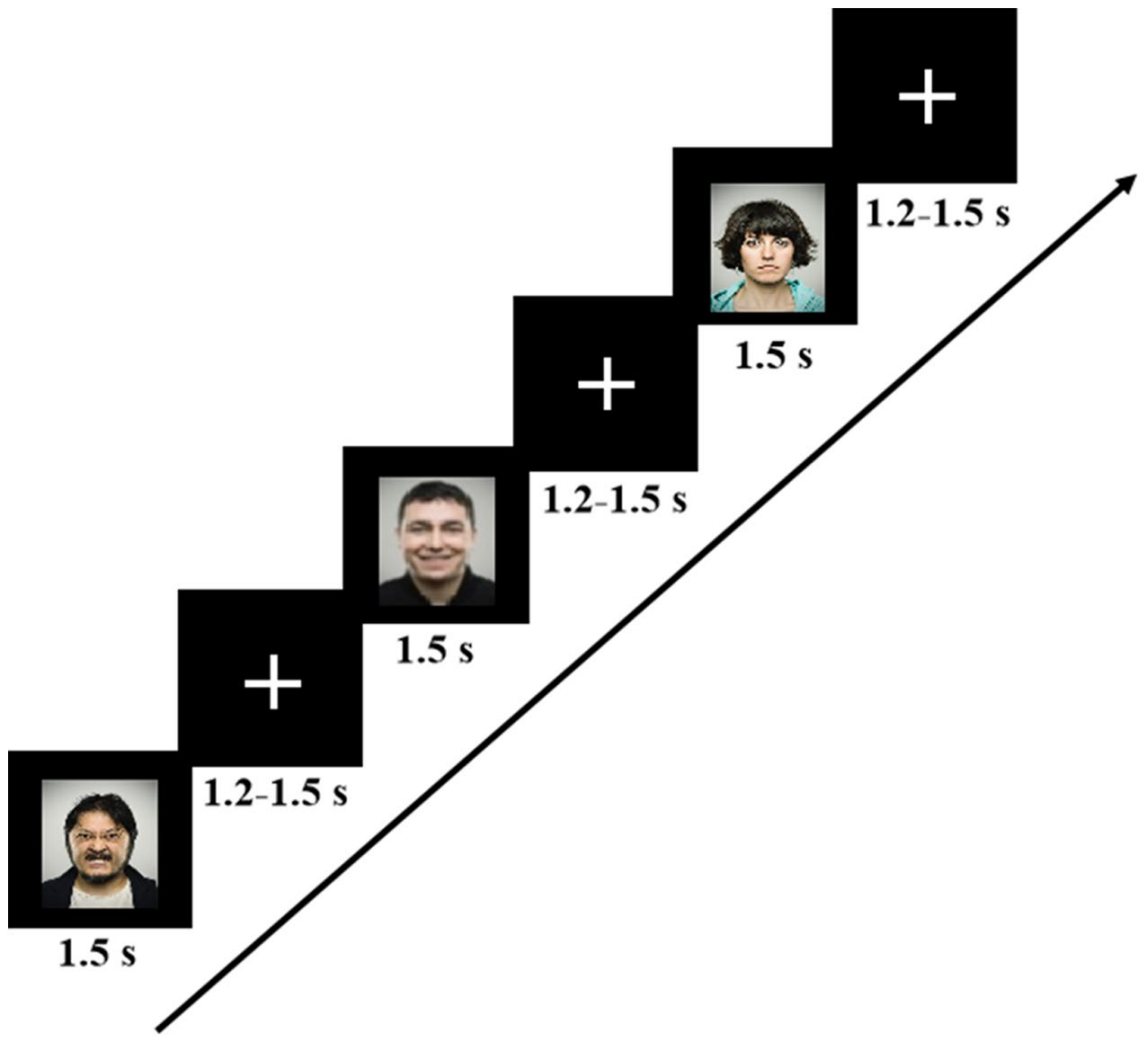
The experimental procedure for the emotional face recognition task. Each image was displayed for 1.5 s, and the interval between images was random, ranging from 1.2 to 1.5 s.

The emotional face recognition task was programmed using 2.0 E-Prime software (Psychology Software Tools, Pittsburgh, PA). Three kinds of facial expressions consisted of stimulus sequence in the task paradigm:60 negative, 60 positive, and 120 neutral faces, with the occurrence probabilities of 25, 25, and 50%, respectively. Each stimulus picture was randomly displayed at the center of the screen on a black background for 1,500 ms, followed by a random inter-stimulus interval of 1,200–1,500 ms. Participants were comfortably seated in a dimly illuminated and sound-attenuated room and viewed the stimuli on a 15-inch LCD screen from a distance of approximately 60 cm. To help participants stay focused, they were instructed to look at the white cross at the center of the monitor and remain as motionless as possible during the inter-stimulus interval. The screen resolution was 72 pixels per inch with a 60-Hz refresh rate, and the viewing angle was 5.8° × 4.6°. The experimental procedure of the task is shown in Figure 1. When a facial expression appeared, subjects were first asked to judge the type of the presented stimulus and then to determine whether they should press a response key or not. They were told to quickly press the key “J” with the right index finger if the positive face appeared on the screen and the key “K” with the right middle finger for the negative face as accurately and fast as possible while ignoring the neutral face. To reduce the interference of muscle movements to EEG signals, subjects were required to remain quiet and, at the same time, move only their right index or middle finger when responding to the stimuli. In addition, a set of facial stimuli, which did not appear in the formal experiment, was used as practice trials for the subjects so that they could be familiar with the experimental process.

### 2.4 EEG acquisition and preprocessing

EEG signals were recorded between 2:00 and 5:00 pm using the Neuroscan Quic-cap device (Neuroscan Inc., Charlotte, NC, USA) from 32 AgCl/Ag scalp electrodes mounted in an elastic cap. The electrodes were arranged according to the international 10–20 system, two of which were placed on the left and right mastoid. The electrode on the right mastoid was used as the reference, which was not shown when recording EEG data. An additional ground electrode was positioned on the forehead. To monitor eye blinks and eye movements, a pair of bipolar electrodes positioned on the supra- and sub-orbit of the left eye and another pair positioned slightly lateral to the outer canthus of each eye simultaneously recorded the vertical and horizontal electrooculogram. During the entire signal acquisition process, the impedance of each electrode was maintained below 5 kΩ. EEG data were continuously acquired at a sample rate of 1,000 Hz with a band-pass of 0.05–400 Hz online.

After recording EEG data, the preprocessing of data was used in the EEGLAB toolbox (Delorme and Makeig, 2004) implemented in the MATLAB R2020a software. First, some electrodes, including the left mastoid electrode and vertical and horizontal electrooculogram electrodes, that were not used in subsequent data analysis were excluded. Second, EEG data were band-pass filtered offline at 1–30 Hz to eliminate low-frequency drift and high-frequency physiological interference. Third, independent component analysis (ICA) was adopted to identify and reject artifacts such as eye blinks and movements. Then, data were re-referenced against the common average reference. Subsequently, we segmented data into epochs time-locked to stimulus onset for each subject. The epoch was defined as the time period from 200 ms before stimulus onset until 900 ms after stimulus onset, and the first 200 ms as a baseline window was used for baseline correction. The epochs with artifacts (amplitude exceeding ±70 μV) or incorrect responses were rejected and excluded from the analysis. Finally, epochs were redefined with the rejection of the first 200 ms for subsequent analysis. The available epochs for the HC and MDD groups were 44.44 ± 10.00 and 43.5 ± 10.18 for negative facial stimuli and 42.25 ± 7.45 and 40.63 ± 9.14 for positive facial stimuli, respectively. There are no significant differences in the number of epochs between the two groups for negative and positive faces, respectively (*p* > 0.05). Additionally, EEG data for all subjects were visually carefully examined after the ICA process. It was found that some subjects had poor-quality EEG in the prefrontal area (i.e., FP1 and FP2), while some subjects had poor-quality EEG in the occipital area (i.e., O1, Oz, and O2). To make the analyzed EEG data consistent for each subject, these five electrodes were also excluded. Therefore, 25 electrodes (i.e., F7, F3, Fz, F4, F8, FT7, FC3, FCz, FC4, FT8, T7, C3, Cz, C4, T8, TP7, CP3, CPz, CP4, TP8, P7, P3, Pz, P4, and P8) were ultimately left to use for further data analysis.

### 2.5 Global field power analysis

Despite most cognitive tasks occurring on a very short duration (usually as short as a fewer hundred milliseconds) (Başar-Eroglu and Demiralp, 2001; Wang et al., 2016; Rizkallah et al., 2018; Fang et al., 2020), the whole cognitive process can be decomposed into a series of stages involving the transient-stable functional brain networks. Furthermore, each stage corresponding to the specific neuropsychological process of the cognitive task has its own start and end time. Therefore, these different stages should be strictly distinguished by employing appropriate methods. In the present study, global field power (GFP) was applied to evaluate the state of global brain activity (Lehmann and Skrandies, 1980), which is defined as the squared root of the mean of the squared potential difference between each EEG electrode (i.e., $y_{i}\left(t\right)$ ) and the mean potential across all *N* electrodes (i.e., $\bar{y\left(t\right)}$ ):


(1) $$GFP\left(t\right)=\sqrt{\left(\overset{N}{\underset{i=1}{\sum }}{\left(y_{i}\left(t\right)-\bar{y\left(t\right)}\right)}^{2}\right)/N}$$

Where $y_{i}\left(t\right)$ is the EEG potential at the *i*-th electrode and $\bar{y\left(t\right)}=\overset{N}{\underset{i=1}{\sum }}y_{i}\left(t\right)/N$ is the mean potential of all electrodes. GFP stands for the strength of the electric field generated by brain activity over the brain at each moment in time, and it is commonly used as an effective way to measure the global brain response to an event or a stimulus (Khanna et al., 2015). Local maxima in the GFP curve with the highest signal-to-noise ratio imply the strongest global field strength at corresponding time instants (Khanna et al., 2015). Therefore, in the present study, the negative peaks (i.e., local minima) of the GFP curve were regarded as the transitions of different stages during emotional face recognition.

### 2.6 Phase transfer entropy

After the cognitive task was divided into different stages, the connections between brain regions at each stage were estimated by PTE (Lobier et al., 2014). The PTE approach is based on the same principles as Wiener−Granger causality, which includes the fact that a source signal has a causal influence on a target signal. This could improve the ability to predict the target’s future if knowing the past of both signals is compared to knowing only the target’s past. In the framework of information theory, this can be best understood in the uncertainty concept: the uncertainty of the target signal determined by its own past and the source signal’s past will be lower than that of determining it only on its own past. In the study, 25 electrodes were employed for PTE calculation. Given a signal *X*(*t*), the analytical signal *A*(*t*) of *X*(*t*) is defined as:


(2) $$A\left(t\right)=X\left(t\right)+iX_{h}\left(t\right)=S\left(t\right)e^{i\theta \left(t\right)}$$

where *S*(*t*) and *θ*(*t*) are the instantaneous amplitude and phase of the analytical signal *A*(*t*), respectively. The function $X_{h}\left(t\right)$ represents the Hilbert transform of *X*(*t*), which is expressed as follows:


(3) $$X_{h}\left(t\right)=\frac{1}{\pi }P.V.\overset{+\infty }{\underset{-\infty }{\int }}\frac{X\left(t\right)}{t-\tau }d\tau$$

where P.V. indicates the integral taken in the sense of the Cauchy principle value (Le Van Quyen et al., 2001).

Once the instantaneous phases of signals *X*(*t*) and *Y*(*t*) were obtained, the PTE from signal *X*(*t*) to signal *Y*(*t*) can be formulated as (Lobier et al., 2014):


4) $$\begin{array}{l} PTE_{X\to Y}\left(t\right)=H\left(\theta_{y}\left(t\right),\theta_{y}\left(t^{\prime }\right)\right)+H\left(\theta_{y}\left(t^{\prime }\right),\theta_{x}\left(t^{\prime }\right)\right)\\ -H\left(\theta_{y}\left(t^{\prime }\right)\right)-H\left(\theta_{y}\left(t\right),\theta_{y}\left(t^{\prime }\right),\theta_{x}\left(t^{\prime }\right)\right) \end{array}$$

where $\theta_{x}\left(t^{\prime }\right)$ and $\theta_{y}\left(t^{\prime }\right)$ denote the past states of the instantaneous phase time series of *X*(*t*) and *Y*(*t*) at $t^{\prime }=t-\delta$ , respectively. $H\left(•\right)$ is Shannon entropy and *δ* is the time lag.

Since the PTE does not have a meaningful upper bound and to reduce biases (i.e., a small non-zero PTE value may mean no actual influence from a source signal to a target signal), we further normalized the PTE by the way Hillebrand et al. (2016) used:


(5) $$dPTE_{X\to Y}\left(t\right)=\frac{PTE_{X\to Y}\left(t\right)}{PTE_{X\to Y}\left(t\right)+PTE_{Y\to X}\left(t\right)}$$

### 2.7 Network analysis

After computing the PTE values between all pairs of EEG electrodes, a weighted directed connectivity matrix was established for each stage for each subject. According to graph theoretical analysis, the human brain is considered a complex network mathematically described as a graph. All matrices were then converted into weighted networks with nodes representing electrodes and edges representing connection strength between each pair of nodes. It should also be noted that the differences in network characteristics had substantial significance for two compared networks with the same number of links (i.e., the connection density of two networks is equal) (Jalili, 2016). On the other hand, the brain network should be sparse to ensure that it is characterized by non-trivial topological attributes such as high network efficiency (Wang et al., 2021). Since there is no uniform standard to determine an optimal threshold, any single threshold is unacceptable, and the topology of the resulting network may uniquely depend on the precise threshold value. Therefore, in the current study, a series of threshold values were chosen to make constructed graphs satisfy the following two conditions: (1) the mean degree (i.e., number of links connected to each node) should be greater than 2ln (*N*) to minimize the number of spurious edges in each network (Achard et al., 2007), where *N* denotes the number of nodes in the network, and (2) the connection density should be lower than 0.5 to ensure sparse properties of networks. Based on these two criteria, we ultimately selected a set of threshold values ranging from 0.275 to 0.495 with an interval of 0.005 through calculation.

Many measures were proposed to characterize the topological properties of a network (Rubinov and Sporns, 2010). In this study, we chose the four most commonly used global network parameters to describe the features of brain network topology at different stages during emotion processing. Characteristic path length (*L*) and clustering coefficient (*C*) quantify the ability of a network to efficiently integrate and process information at global and local levels, respectively (Watts and Strogatz, 1988). These two metrics correspond to two basic brain functional principles: integration and segregation. *L* is the average of the shortest path length between any pair of nodes. The *C* of a node is quantified by the ratio of the number of existing triangles in the node to the maximum number of possible triangles around the node. The *C* of the entire network is the average clustering coefficient of all nodes with a value varying between 0 and 1. To investigate the small-worldness attributes of a network, normalized characteristic path length $\lambda =L/L_{\mathrm{rand}}$ and normalized clustering coefficient $\gamma =C/C_{\mathrm{rand}}$ were calculated as a function of threshold (Watts and Strogatz, 1988), where *L_rand_* and *C_rand_* are the values of *L* and *C* derived from the matched random network. The randomized networks preserved the same nodes, edges, and degree distribution in the original network (Maslov and Sneppen, 2002). In the present study, 100 matched random networks were generated, and *L_rand_* and *C_rand_* were then obtained by averaging 100 groups of random networks for comparison with the original networks. It has been proven that a small-worldness network was characterized by the values of *γ*>1 and *λ* ≈ 1 (Watts and Strogatz, 1988). For regional nodal metrics, betweenness centrality (*BC*) was selected to examine the regional characteristics of the brain networks. *BC* is defined as the fraction of all shortest paths in the network that pass through a given node, which reflects the influence of the node in the whole network. In addition, we also evaluated the asymmetry index of the network parameter *BC* by the formula: $\mathrm{AI}=\left(L_{BC}-R_{BC}\right)/\left(L_{BC}+R_{BC}\right)$ , where *L_BC_* and *R_BC_* represent the average *BC* of the left and right hemispheres, respectively. Positive *AI* value indicates leftward asymmetry and vice versa.

In this study, a freely available Brain Connectivity Toolbox (BCT) was applied for both the calculation of all network metrics and the construction of random networks (Rubinov and Sporns, 2010). For each threshold value (threshold values varied from 0.275 to 0.495, and the interval was 0.005), the elements in the connectivity matrix whose values were less than the threshold value were assigned zero values, and the other elements remained unchanged. Then, the corresponding network metrics, including *L*, *C*, *BC*, *L_rand_*, and *C_rand_* can be calculated by BCT. Of course, *λ = L/L_rand_* and *γ = C/C_rand_* can also be obtained. To quantitatively compare the differences in brain network topology between MDD and HC groups, the integrated network parameters (*L*, *C*, *BC*, *λ*, and *γ*) were computed for each stage for each subject in accordance with the approach by Tian et al. (2011), which was defined as: $X=\underset{k}{\sum }X\left(k\right)\Delta k$ , where *k* is a threshold value, *X*(*k*) is the network metric under the threshold value *k* condition, and Δk represents the threshold interval. In addition, based on the idea that central nodes acting as key roles are involved in lots of short paths within a network (Freeman, 1978), *BC* was also employed to identify the hub nodes in the brain network after computing the normalized parameter of each node: a node was identified as a hub only if its *BC* value was at least larger than the sum of the standard deviation and the mean across *BC* values of all nodes (Tian et al., 2011).

### 2.8 Statistical analysis

To determine the significant differences in brain network measurements between MDD patients and normal subjects in each case, the SPSS Statistics 25 software (IBM, Armonk, NY, USA) was applied in this study. First, the normal distributions of the two groups of data were examined using the Kolmogorov–Smirnov test. If both the data satisfied normal distributions, the differences between the two groups of data were analyzed by performing an independent samples *t*-test; otherwise, the Mann–Whitney *U*-test was used. Repeated measures analysis of variance (ANOVA) was used to determine whether there were significant differences in integrated network metrics (i.e., *L*, *C*, *λ*, and *γ*) between the MDD group and the HC group. Three factors were included: between-subject factor *Group* (two levels: MDD/HC groups), within-subject factor *Emotion* (two levels: positive/negative emotion conditions), and within-subject factor *Stage* (three levels: stages 1, 2, and 3). The Greenhouse–Geisser correction was employed to correct value of *p*s when Mauchly’s test of sphericity was invalid. The obtained value of *p*s were adjusted by false discovery rate (FDR) for multiple comparisons. If there were significant differences between the two groups in any network metrics, the relationships between HAMD scores in patients with MDD and these metrics were further investigated by the linear regression method. Additionally, a value of *p* less than 0.05 was considered to be statistically significant.

## 3 Results

### 3.1 Behavioral results

The performance of response time and accuracy rate for HC and MDD groups in the emotional face recognition task is presented in Table 2. From the table, it can be seen that patients with MDD showed longer response time of facial expressions recognition related to the normal group, and there was a significant difference between the two groups for negative face condition (independent samples *t-*test, *t* = 2.590, effect sizes = 5.035, *p* = 0.014), indicating that the depressed individuals needed to spend more time on the emotional face processing. In terms of accuracy rate, the performance of both positive and negative face identification for the MDD group was relatively lower than that for the HC group, which may reflect the dysfunctional emotion processing of MDD patients. Moreover, the results also revealed that compared to normal subjects, a significantly lower accuracy rate of neutral face recognition was observed in the MDD group (the Mann–Whitney *U-*test, *U* = 28, effect sizes = −8.155, *p* = 0.000).

**Table 2:** The statistical results of behavioral data between HC and MDD groups for emotional face recognition task.

Face stimulus type	Behavioral index	HC group (mean ± SD)	MDD group (mean ± SD)	p
Negative	Response time (ms)	698.39 ± 67.35	771.17 ± 95.52	0.014^c^
	Accuracy rate (%)	96.48 ± 2.74	92.19 ± 7.59	0.102^b^
Positive	Response time (ms)	670.08 ± 82.33	719.68 ± 112.91	0.224^b^
	Accuracy rate (%)	97.13 ± 3.74	93.33 ± 10.35	0.403^b^
Neutral	Accuracy rate (%)	97.87 ± 4.00	79.58 ± 18.04	0.000^b^

^b^Mann-Whitney *U*-test; ^c^Independent samples *t*-test; HC, healthy control; MDD, major depressive disorder.

### 3.2 Global field potential analysis results

**Figure 2:**
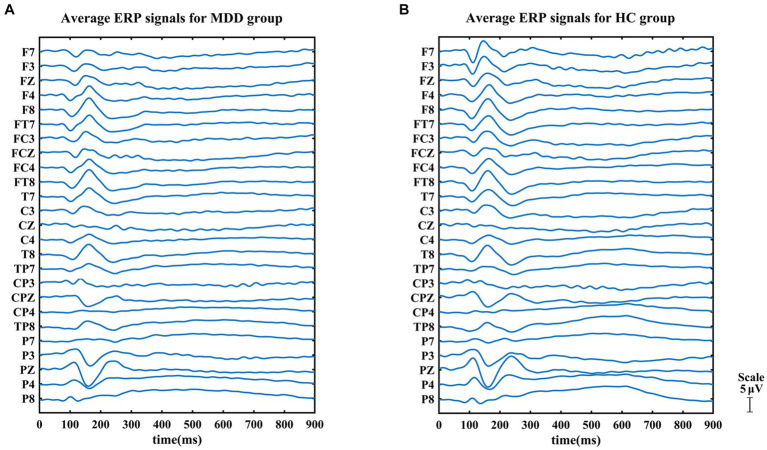
Event-related potential (ERP) signals for negative face recognition task: **(A)** MDD group and **(B)** HC group. We can observe that the ERP signals of the two groups were different, especially in the frontal area.

**Figure 3:**
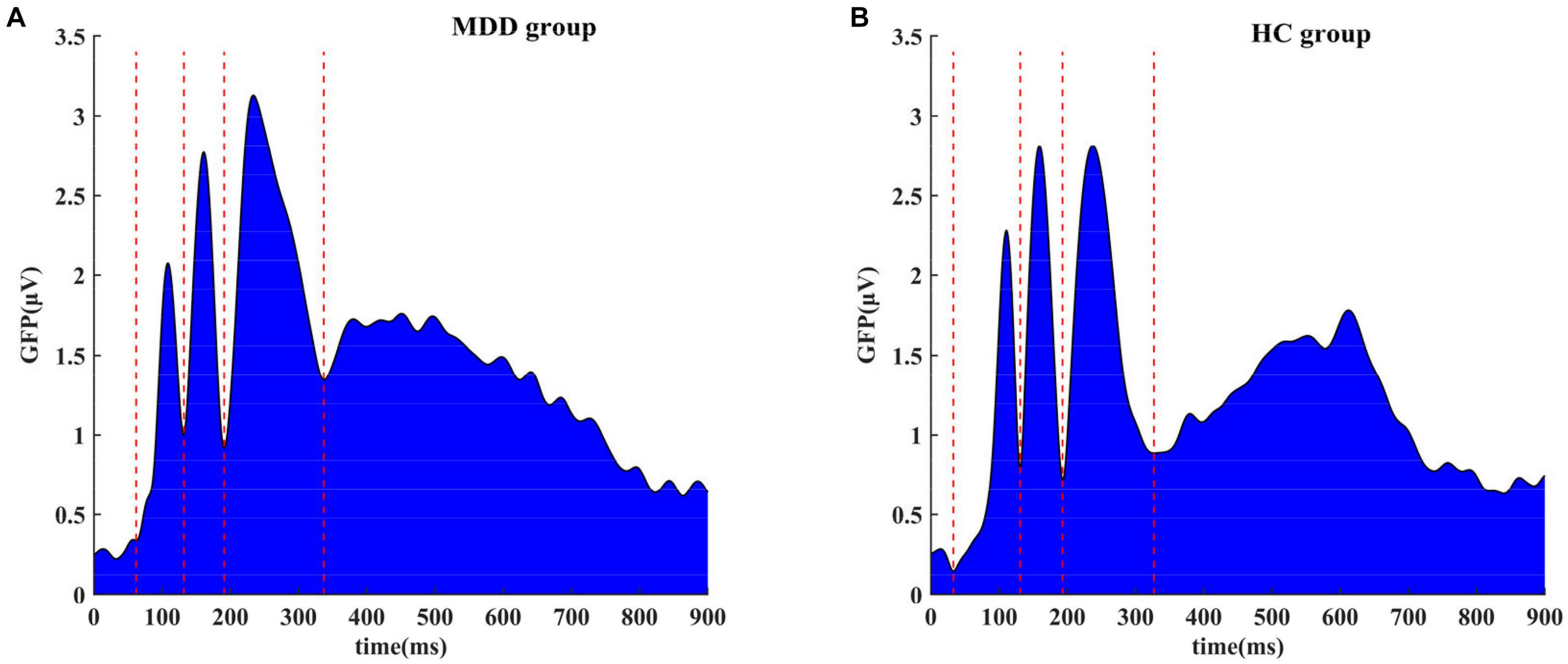
Global field potential (GFP) of group-averaged event-related potential (ERP) signals for negative face recognition task: **(A)** MDD group; **(B)** HC group. The process was divided into several stages for the two groups according to the local minima of GFP.

By averaging negative stimulus epochs, the group-averaged ERP signals were obtained for the normal and depressed groups (Figure 2). For the sake of visual comparison, a superimposing of both groups’ results for the individual channel was also presented (Supplementary Figure S1). The GFP method was then employed to analyze group-averaged ERP signals for each group to identify different stages during the task. The results of the GFP analysis for the negative face recognition task are shown in Figure 3. From the figure, we can see that the process was divided into multiple stages for both two groups. Similar results for the positive face recognition task were also found in the normal and MDD groups (see Supplementary Figures S2–S4). Considering all the cognitive processes involved in the whole task, the last phase immediately following the third stage should be related to the response stage, namely, subjects responded to stimuli. In this study, we mainly put our interest on the first three stages preceding the response stage, of which the overlapped periods of two groups were focused on for network construction by PTE [i.e., negative face recognition: stage 1 (64–131 ms), stage 2 (132–193 ms), and stage 3 (194–323 ms); positive face recognition: stage 1 (63–129 ms), stage 2 (130–189 ms), and stage 3 (192–332 ms)]. Furthermore, the decision-making stage should precede the response stage, so stage 3 for both HC and MDD groups was very likely to be the stage of emotional decision-making.

### 3.3 Network analysis results

**Figure 4:**
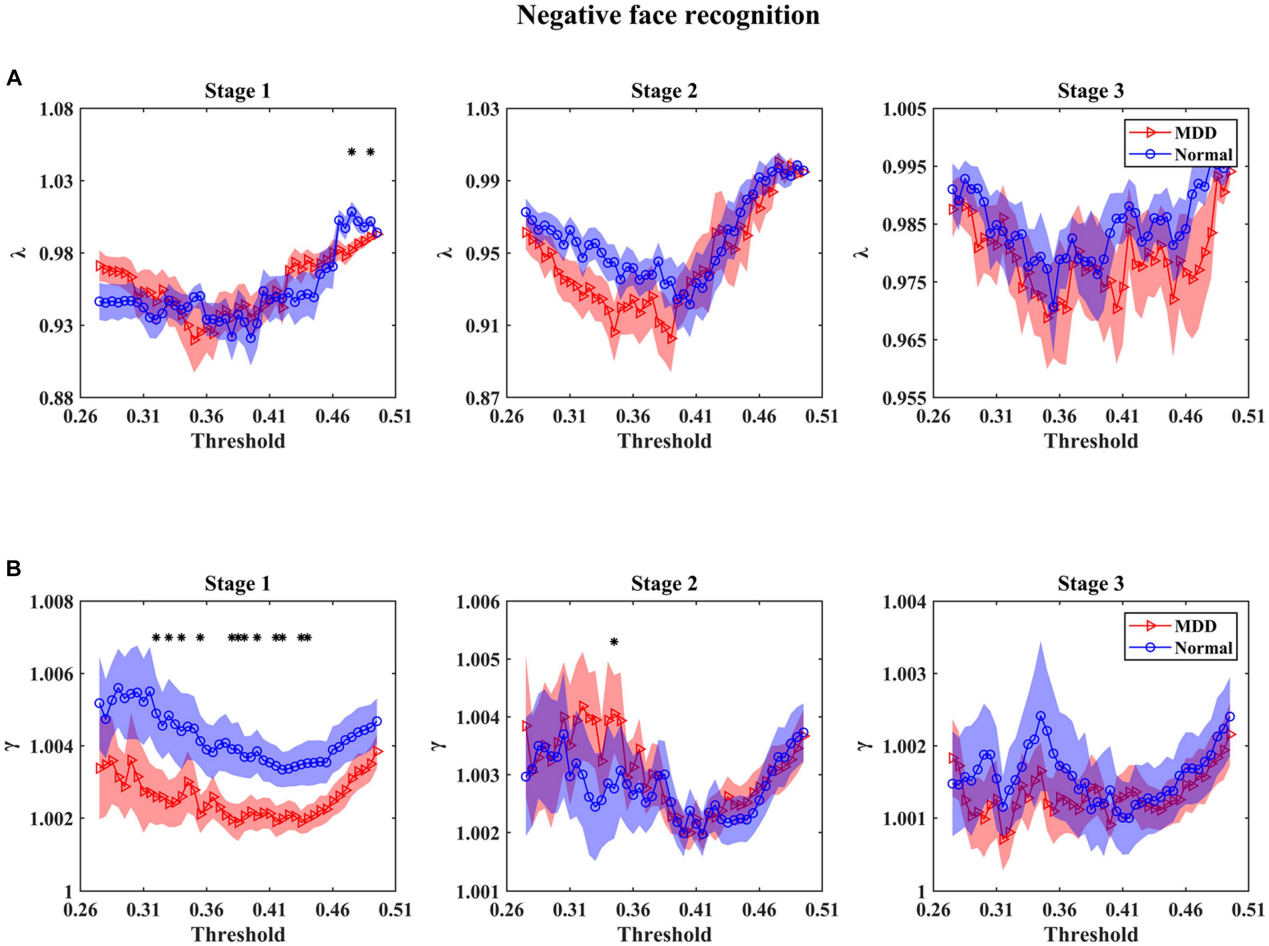
Mean values of small-world parameters as a function of the threshold for normal controls and patients with MDD at different stages under negative face recognition conditions. *λ* and *γ* denote normalized characteristic path length **(A)** and normalized clustering coefficient **(B)**, respectively. Shaded areas around the mean values denote the standard errors. The asterisks indicate significant differences between the MDD and normal groups (*p* < 0.05). The main finding we found was that the *γ* values of MDD patients were significantly smaller than that of normal controls under multiple thresholds at stage 1 of negative face processing.

**Figure 5:**
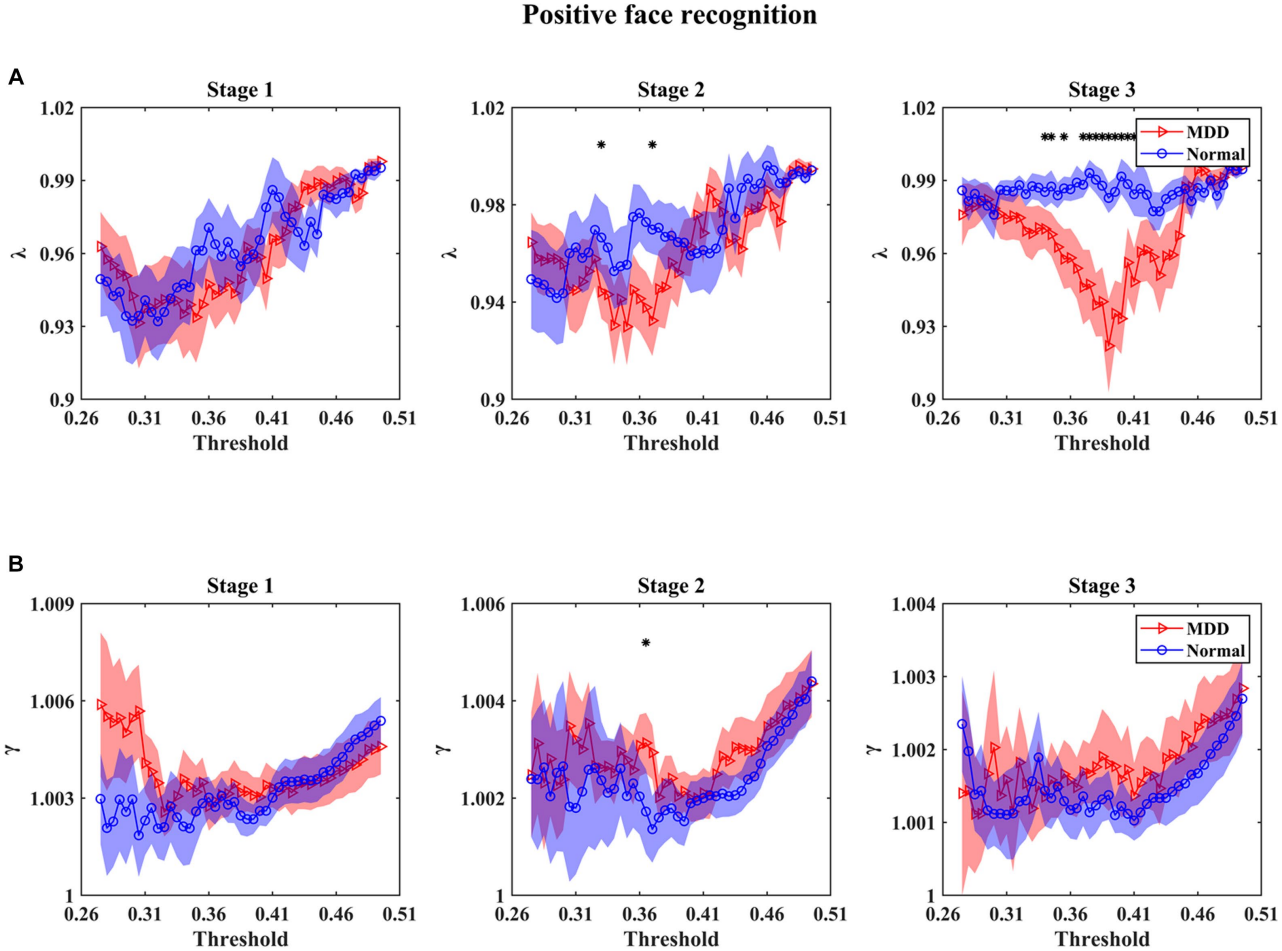
Mean values of small-world parameters as a function of the threshold for normal controls and patients with MDD at different stages under positive face recognition conditions. *λ* and *γ* denote normalized characteristic path length **(A)** and normalized clustering coefficient **(B)**, respectively. Shaded areas around the mean values denote the standard errors. The asterisks indicate significant differences between the MDD and normal groups (*p* < 0.05). The main finding we found was that the *λ* values of MDD patients were significantly smaller than that of normal controls under multiple thresholds at stage 3 of positive face processing.

For small-world parameters (i.e., *λ* and *γ*) analysis as a function of threshold at three stages in the emotional face processing, the results in Figures 4, 5 show that the values of *γ* in functional brain networks for two groups were larger than 1 (i.e., the *C* of these networks were larger than those of the matched random networks), while the values of *λ* for both groups tended to the value 1 (i.e., the *L* of these networks were comparable to those of the matched random networks), manifesting that both of the depressed and normal groups presented the characteristics of a small-world network at three stages during facial expression recognition. In particular, we mainly found that (1) MDD patients had statistically significant decrease of *γ* for multiple threshold values at the first stage under negative face condition relative to healthy subjects (Figure 4) (for statistical details, see Supplementary Table S1); (2) For positive face identification, as shown in Figure 5, the values of *λ* for the HC group were almost significantly higher than that for the MDD group over the threshold range of 0.34–0.41 at the third stage (for statistical details, see Supplementary Table S2). Nevertheless, there was almost no significant difference in *γ* and *λ* between the two groups at other threshold values for both negative and positive emotion conditions. Therefore, the results stated that the functional brain networks of patients with MDD tended to have a randomization in the processing of facial expressions compared to normal individuals. Furthermore, there were no significant *Group* and *Emotion* effects on any of the four integrated global network parameters (*p >* 0.05), and none of the interactions of *Group*×*Emotion*, *Group*×*Stage*, *Emotion*×*Stage*, and *Group*×*Emotion*×*Stage* reached significance (*p >* 0.05). However, a significant *Stage* effect was observed in all global network metrics but the parameter *γ* (*L*: df = 2, *F* = 7.013, *p* = 0.001; *C*: df = 2, *F* = 14.863, *p* = 0.000; *λ*: df = 2, *F* = 13.850, *p* = 0.000). A further simple effect analysis was performed afterward.

**Figure 6:**
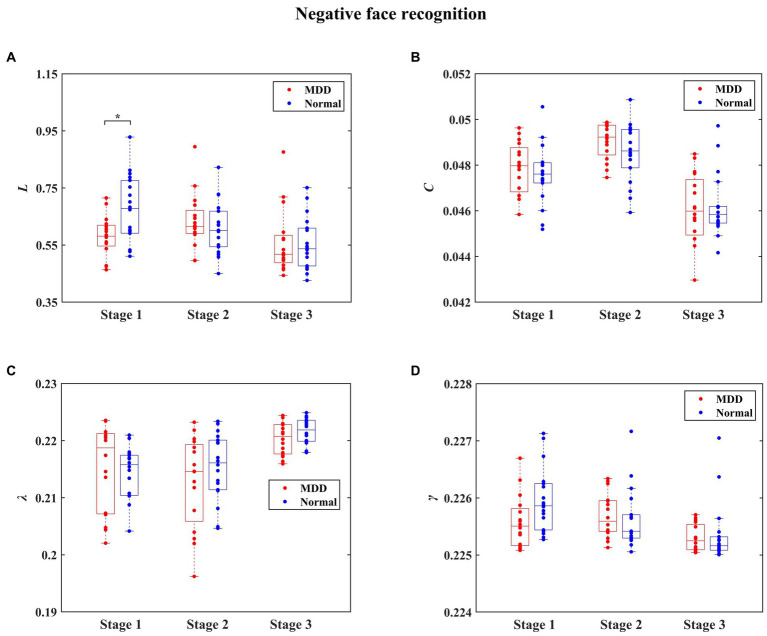
The statistical results of integrated network metrics for the normal and depressed groups at different stages during negative face recognition. The integrated network metrics *L*, *C*, *λ,* and *γ* denote characteristic path length, clustering coefficient, normalized characteristic path length, and normalized clustering coefficient, respectively. We found that compared to the normal group, *L* was significantly decreased in the MDD group (* denotes *p* < 0.05, FDR correction).

**Figure 7:**
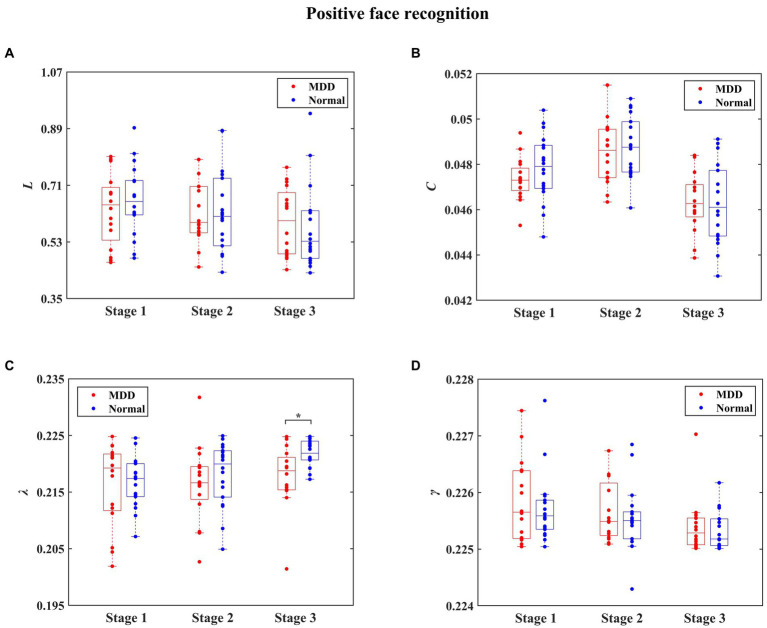
The statistical results of integrated network metrics for the normal and depressed groups at different stages during positive face recognition. The integrated network metrics *L*, *C*, *λ,* and *γ* denote characteristic path length, clustering coefficient, normalized characteristic path length, and normalized clustering coefficient, respectively. We found that compared to the normal group, *λ* was significantly decreased in the MDD group (* denotes *p* < 0.05, FDR correction).

The statistical results of integrated network metrics between normal people and MDD patients for three stages during negative face recognition are shown in Figure 6. Significantly decreased *L* in MDD patients was found at the first stage for negative face condition relative to the normal group (independent samples *t*-test, *t* = −2.832, effect sizes = −5.350, *p* = 0.024, FDR correction). Moreover, it can be seen in Figure 7 that the MDD group exhibited a remarkably smaller *λ* value than healthy individuals at the third stage in the process of positive face identification (independent samples *t*-test, *t* = −2.830, effect sizes = −0.384, *p* = 0.024, FDR correction). Apart from these two findings, we did not find any significant differences between the two groups at other stages for the emotional face discrimination task.

**Figure 8:**
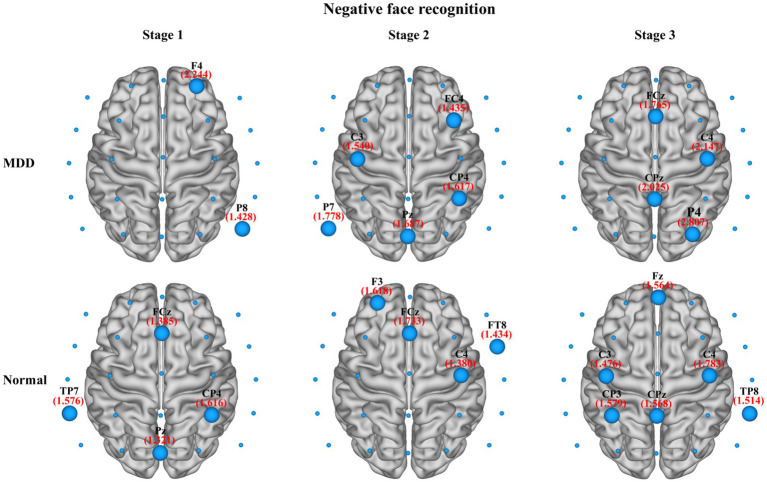
The identified network hubs and their betweenness centrality (BC) values for the MDD and normal groups at different stages under negative face recognition conditions. For each stage, the distribution and size of core hub nodes were different between the two groups, suggesting that there may be functional abnormalities for MDD patients in negative emotional processing.

**Figure 9:**
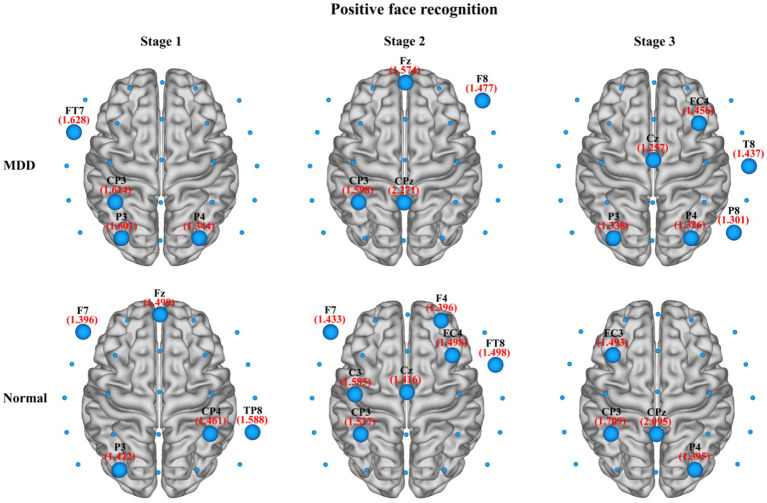
The identified network hubs and their betweenness centrality (BC) values for the MDD and normal groups at different stages under positive face recognition conditions. For each stage, the distribution and size of core hub nodes were different between the two groups, suggesting that there may be functional abnormalities for MDD patients in positive emotional processing.

In the present study, we also compared the average nodal *BC* in functional brain networks of depression patients and healthy people at different stages of emotional face processing (see Table 3 for details). The table shows an observably smaller *BC* value in patients with MDD at the first stage of the negative face information processing compared to the normal group (independent samples *t*-test, *t* = −2.934, effect sizes = −5.504, *p* = 0.021, FDR correction). However, we failed to find any significant differences in asymmetry for each stage of the emotional face recognition task. Moreover, regarding network hubs, Figures 8, 9 present hub nodes and their *BC* values, respectively, at three stages during negative and positive face recognition [3D brain networks were visualized using the BrainNet Viewer Toolbox developed by Xia et al. (2013)]. The results indicated that at stage 1, the hub nodes of the networks in both normal and depressed groups for negative and positive face identification involved the parietal brain region, including P3, P4, P8, and Pz, and the central-parietal region (CP3) was not a hub in the brain network of MDD patients under negative face condition; at stage 2, the distribution of hubs for two groups during emotion processing was different except for the common hub (CP3) for positive face condition. At stage 3, both groups’ brain networks showed the same hubs (C4 and CPz) in the negative face processing; the P4 was identified as a hub node for healthy people and MDD patients during positive face recognition. Additionally, for both two conditions, the CP3 was a hub only for the brain network of the HC group.

**Table 3:** Average nodal betweenness centrality (BC) of functional brain networks for MDD patients and normal subjects at different stages in the process of emotional face recognition.

Emotional face recognition	Stage 1	Stage 2	Stage 3
Negative	1.34 ± 0.66	1.92 ± 1.09	1.07 ± 1.12
	2.36 ± 1.29	1.67 ± 0.95	1.01 ± 0.78
Positive	1.88 ± 1.12	1.86 ± 0.95	1.39 ± 1.02
	2.22 ± 1.22	1.94 ± 1.42	1.24 ± 1.23

For each face type, the upper and lower rows denote the values of the MDD and normal groups, respectively. The bold number presents the significant difference between the two groups (*p* < 0.05, FDR correction).

### 3.4 Relationships between network metrics and HAMD scores

**Figure 10:**
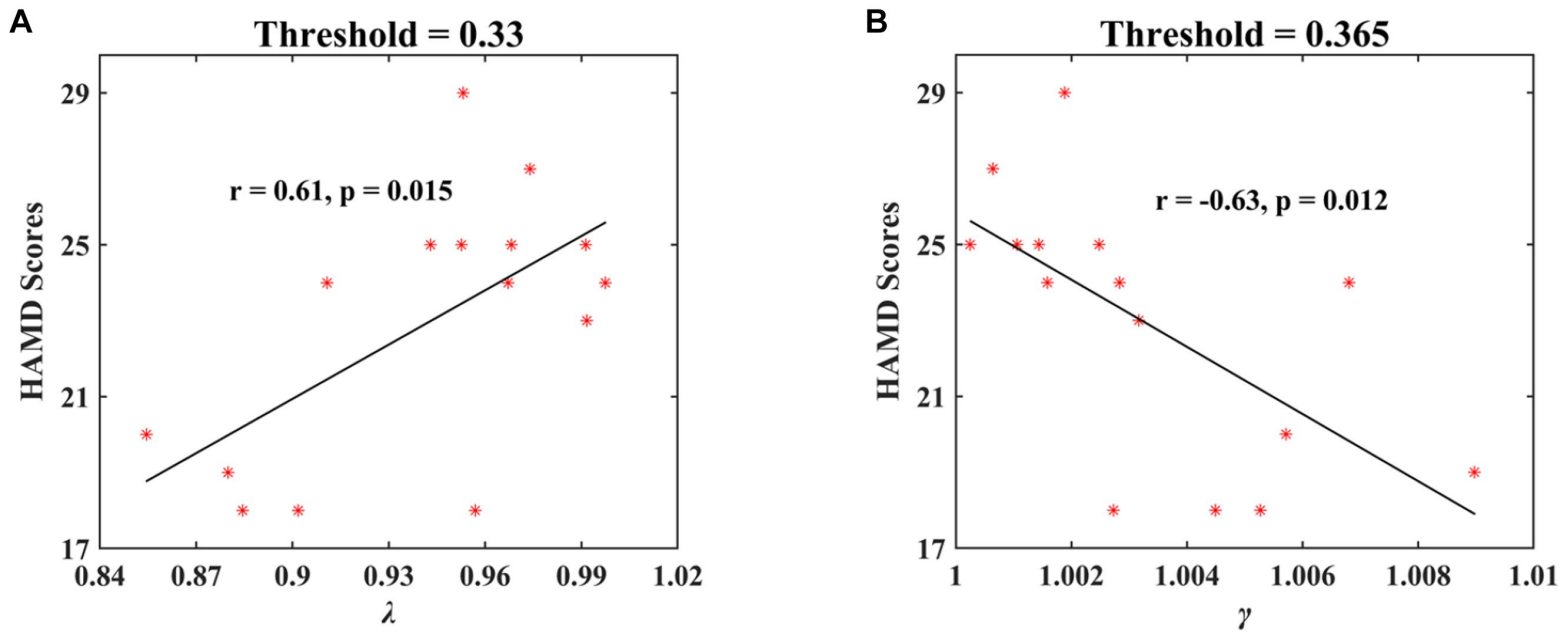
The significant correlations of HAMD scores in MDD patients and normalized characteristic path length (λ) and normalized clustering coefficient (*γ*) at the second stage of positive face recognition. The results showed a significant positive correlation between λ and HAMD scores and a significant negative correlation between *γ* and HAMD scores.

For the significant network metrics between the MDD and HC groups identified above, the relationships of these metrics and HAMD scores in MDD patients were further analyzed by using linear regression approach. The significant correlations were observed at the third stage of positive face identification under multiple threshold conditions. The λ showed significantly positive correlation with HAMD scores when threshold was 0.33 (*r* = 0.61, *p* = 0.015; Figure 10A). There was significantly negative relationship between γ and HAMD scores when threshold was 0.365 (*r* = −0.63, *p* = 0.012; Figure 10B). No significant relationships between network metrics and HAMD scores of depression severity were found in other cases.

## 4 Discussion

To the best of our knowledge, this study is the first attempt at investigating the differences in topological properties of functional brain networks between patients with MDD and healthy subjects at different stages during emotional face recognition by using EEG through graph theoretical approaches. First, the facial expression recognition task was divided into several stages by GFP, and each stage corresponded to a specific phase of emotional face information processing. Then, the three successive stages preceding the response stage in which subjects responded to face stimuli were selected for further network analysis. Our results revealed that the functional brain networks of both normal and MDD groups exhibited the small-world features for three stages of facial recognition, whereas MDD patients’ brain networks showed a tendency to be randomized through the comparison of normalized characteristic path length and normalized clustering coefficient between the two groups. It was also found that compared to healthy subjects, the MDD group had significantly lower characteristic path length at the first stage under negative face conditions and normalized characteristic path length at the third stage of positive face recognition, respectively. Moreover, the central-parietal region may lose its role as a core node in emotional processing for MDD patients.

Numerous previous studies have demonstrated that the procedure of a cognitive task can be decomposed into a sequence of distinct stages, each of which associates with a transiently stable neuropsychological state characterized by its corresponding specific functional brain network (Karamzadeh et al., 2013; Wang et al., 2016; Fang et al., 2020). Because similar topographies during the periods within several tens of milliseconds were disrupted by brief peaks (i.e., topographic map changes), the GFP peaks were employed to quantify the stability of the maps over time (Michel et al., 2004). Relative to other approaches [e.g., ERP segmentation was based on the selection of the expected latency windows according to previous experience (Karamzadeh et al., 2013)], the GFP technique has the advantages of being more objective and convenient. Furthermore, a peak in the GFP signal is generally considered to be a stable state, which has been extensively used for EEG microstate analysis (Yoshimura et al., 2019; Pan et al., 2020; Li et al., 2021; Zhao et al., 2022). Therefore, in the present study, we utilized the peaks of GFP curves calculated from group-averaged ERP signals to determine each stage to explore the topological attributes of brain networks during facial expression identification.

In this study, at stage 1 during emotional face recognition, the results indicated that the functional brain networks of both normal and depressed groups had comparable characteristics to the small-world network, but there existed significant group differences in small-world and integrated network metrics. Specifically, MDD patients showed significantly reduced normalized clustering coefficient and characteristic path length in negative face processing compared to healthy controls, supporting abnormal alterations in local information processing efficiency and global information integration (Shim et al., 2018). Given that the human brain is modeled by the small-world topology in support of both specialized and distributed information processing, random networks comprise relatively shorter characteristic path lengths and smaller clustering coefficients than small-world networks. Therefore, these findings reflected a disruption of the normal balance between functional segregation and functional integration in the brain network of MDD, which suggested that the depression patients’ brain network was closer to a randomized configuration. Research based on intracranial or scalp EEG has shown that the oscillatory response induced by stimulus occurred within 150 ms (Karakaş and Basar, 1998; Schendan et al., 1998; Oya et al., 2002), which covered the duration of brain networks for two groups at this stage. Furthermore, in line with our findings, another two studies revealed that the duration of the brain network involved in the visual perception stage also appeared roughly in this time range (Hassan et al., 2015; Fang et al., 2020). Although the two groups’ brain networks were different for this stage of emotion processing in terms of hub nodes, there was a commonality, namely, one or two of the identified hubs belonged to the parietal region, such as P3, P4, Pz, and P8, indicating that the parietal region was closely connected with multiple other brain regions and acted as a critical role in information exchange between brain regions. As is well-known, the parietal region is associated with the somatosensory cortex and adjacent to the visual cortex area. It was also found that there were visual perception areas in the parietal cortex when decoding facial stimuli (Sereno and Huang, 2006). Therefore, we suspected that the functional brain network of the first stage was involved in visual perception.

At stage 2 of the negative and positive face information processing, we found little significant difference between the normal and depressed groups with regard to network metrics. The duration of brain networks at the second stage of emotional face identification (negative: 132–193 ms; positive: 130–189 ms) contained a latency of N170 (about 130–200 ms), which was induced by facial presentation and correlated highly with the neurophysiological mechanisms underlying facial recognition (Rossion, 2014; Damaskinou and Watling, 2018; Lai et al., 2020; Schindler et al., 2021). Therefore, this stage probably belonged to the face recognition stage. Since N170 is an exogenous component, it only depends on face stimuli and has nothing to do with the subjective arousal state of subjects. Therefore, this may be an important reason why there was almost no significant difference between the two groups for global brain network characteristics at this stage. However, this finding seemed to be inconsistent with many previous studies, which have found differences in N170 between patients with depression and controls. Because the sample size was relatively small in this study, the statistical power of the results was affected. Thus, it is possible that the differences in stage 2 were not statistically significant due to the small sample size of this study. On the other hand, MDD patients and normal individuals had different hub nodes, but each group presented its own specific rules. For the normal group, the identified hubs were mainly located in the frontal, fronto-central, central, and fronto-temporal regions (negative: F3, FCz, FT8, and C4; positive: F7, F4, FC4, FT8, C3, and Cz). For the MDD group, the central, centro-parietal, and parietal regions, including C3, CP4, P7, and Pz, for negative face condition and the frontal (Fz and F8) and centro-parietal (CP3 and CPz) regions under positive face condition were network hubs. The main distinctions between the two groups were that the depressed group’s brain networks lacked frontal and central core nodes for this stage during negative and positive face recognition related to healthy subjects. The prefrontal cortex includes the subgenual prefrontal cortex, medial prefrontal cortex, and dorsolateral prefrontal cortex, and these brain regions are closely associated with the recognition of emotion stimuli and mood regulation (Whittle et al., 2006; Fitzgerald et al., 2008; Price and Drevets, 2012). As one of the main regions in the somatosensory cortex, the precentral cortex responds to sensory and motor processing. Particularly, further studies have confirmed that compared to normal people, depressed patients showed both structural and functional abnormalities in the precentral gyrus (Diener et al., 2012; Wang et al., 2018). Therefore, the dysfunction of the frontal and central areas might be related to the pathophysiological mechanism of MDD.

For functional brain networks at stage 3 (i.e., emotional decision-making stage) in facial expression identification, abnormally decreased normalized characteristic path length was observed in MDD patients during negative face recognition as compared to the normal group, manifesting that the brain network of depression patients had a trend toward a random network. This randomization of functional brain networks has also been found in patients with vascular dementia at the decision-making stage of a visual oddball task (Wang et al., 2016). Together with the above results of the first stage, these findings unequivocally consolidated that the functional brain network in patients with MDD had lost the small-world attributes, which demonstrated that MDD is a disorder with a less optimal network configuration and a deficiency of emotional processing. With regard to hub nodes, Figures 7, 8 display that the normal group had the common network hubs (CP3 and CPz) in the centro-parietal region for both negative and positive face conditions, while only CPz was the hub node in the depressed patients during negative face processing. As the hubs in a brain network played a crucial role in maintaining connectivity and integrating information of the entire network (Hagmann et al., 2008), the finding illustrated that the centro-parietal region served as the primary site of hubs at the decision-making stage of emotion processing for normal subjects, but MDD patients may lose the status of this brain region. Hence, this led to greatly weakened communication between the centro-parietal and other brain regions in the emotional information processing for depressed individuals. In addition, the damaged hubs were considered to be most likely related to the pathology of depression (Zhang et al., 2018).

Particularly, we observed a significant positive correlation between normalized characteristic path length (*λ*) and HAMD scores (threshold = 0.33, *r* = 0.61, *p* = 0.015) and a negative correlation for normalized clustering coefficient (*γ*) (threshold = 0.365, *r* = −0.63, *p* = 0.012) at the second stage of positive face recognition, signifying that depression severity can be predicted by the network metrics *λ* and *γ*. Therefore, the research findings indicated that the small-world attributes, i.e., *λ* and *γ* under positive face recognition task, may serve as a promising electrophysiological biomarker for clinical applications in the diagnosis of MDD.

Some limitations should be addressed in the present study. First, the sample size of this study was relatively small, which affects the statistical power of the study and the generalizability of the results. Therefore, more patients need to be recruited to further validate the findings in future work. Then, the network analysis of this study was based on the scalp EEG level, rather than the source-space level, which may introduce non-neuronal noise and reduce the specificity of the findings, which limited the interpretation of the results. In addition, the default mode network (DMN) and the frontoparietal network (FPN) are closely associated with MDD (Whitton et al., 2017). A separate analysis of these networks could reveal nuanced interactions between different regions and extend our understanding of the pathology of MDD. However, the accuracy of cortical source estimation is largely dependent on sensor density and the head model constructed by individual MRI (Song et al., 2015; Michel and He, 2019). Therefore, we will try to explore the characteristics of cortical brain networks and special networks using a source localization technique by collecting high-density EEG (e.g., 64) and individual MRI for each subject in future work. Additionally, it should be noted that the construction of functional brain networks did not incorporate the occipital electrodes, which were excluded due to poor data quality. Therefore, the results need to be interpreted with caution in the study. In future studies, we will compare the current results with the results without removing the occipital electrodes to validate whether the electrodes in this experiment have an impact on the results or not. Finally, although the emotional stimuli used in the study were not derived from the standardized face databases [e.g., International Affective Picture System (IAPS); Lang et al., 1999], we have validated that these stimuli can correctly elicit emotion in the test subjects.

## 5 Conclusion

In this study, by comparing the topological properties of functional brain networks between normal subjects and MDD patients at different stages during negative and positive face recognition, our results revealed that the constructed functional brain networks for three stages were associated with different neuropsychological phases of facial expressions recognition, including visual perception, face recognition, and emotional decision-making. Significant decreases in characteristic path length at the visual perception stage in the negative face processing and normalized characteristic path length at the emotional decision-making stage during positive face recognition were observed in patients with MDD compared to healthy subjects. Furthermore, the findings suggested that the brain network of MDD patients had a trend to be randomized. In addition, MDD patients may lose the status of the centro-parietal region as a hub in the brain network for emotional information processing. To sum up, the present study revealed abnormalities in the topological properties of the functional brain network of MDD patients during emotional face recognition with insufficient ability to extract facial features and process emotion information, providing new insights into understanding the neural mechanisms of emotional processing dysfunction in MDD from the respective of the functional brain network.

## Data availability statement

The raw data supporting the conclusions of this article will be made available by the authors, without undue reservation.

## Ethics statement

The studies involving humans were approved by the Ethics Committee of the First Affiliated Hospital of Xi’an Jiaotong University. The studies were conducted in accordance with the local legislation and institutional requirements. The participants provided their written informed consent to participate in this study.

## Author contributions

C-LT: Data curation, Formal analysis, Methodology, Visualization, Writing – original draft, Writing – review & editing. LC: Methodology, Visualization, Writing – review & editing. WW: Data curation, Resources, Writing – review & editing. SC: Investigation, Methodology, Writing – original draft. MW: Data curation, Visualization, Writing – original draft. W-TD: Methodology, Writing – original draft. MJ: Data curation, Writing – original draft. JM: Investigation, Methodology, Writing – review & editing, Funding acquisition. JX: Conceptualization, Supervision, Writing – review & editing. W-DH: Investigation, Writing – review & editing, Methodology.

## References

[ref1] Achard S. Bullmore E. Friston K. J. (2007). Efficiency and cost of economical brain functional networks. PLoS Comput. Biol. 3:e17. doi: 10.1371/journal.pcbi.0030017, PMID: 17274684 PMC1794324

[ref2] Ao X. Mo L. Wei Z. Yu W. Zhou F. Zhang D. (2020). Negative bias during early attentional engagement in major depressive disorder as examined using a two-stage model: high sensitivity to sad but bluntness to happy cues. Front. Hum. Neurosci. 14:593010. doi: 10.3389/fnhum.2020.593010, PMID: 33328939 PMC7717997

[ref3] Başar-Eroglu C. Demiralp T. (2001). Event-related theta oscillations: an integrative and comparative approach in the human and animal brain. Int. J. Psychophysiol. 39, 167–195. doi: 10.1016/s0167-8760(00)00140-9, PMID: 11163896

[ref4] Bressler S. L. Menon V. (2010). Large-scale brain networks in cognition: emerging methods and principles. Trends Cogn. Sci. 14, 277–290. doi: 10.1016/j.tics.2010.04.004, PMID: 20493761

[ref5] Brookes M. J. O'Neill G. C. Hall E. L. Woolrich M. W. Baker A. Palazzo Corner S.. (2014). Measuring temporal, spectral and spatial changes in electrophysiological brain network connectivity. NeuroImage 91, 282–299. doi: 10.1016/j.neuroimage.2013.12.066, PMID: 24418505

[ref6] Burkhouse K. L. Owens M. Feurer C. Sosoo E. Kudinova A. Gibb B. E. (2017). Increased neural and pupillary reactivity to emotional faces in adolescents with current and remitted major depressive disorder. Soc. Cogn. Affect. Neurosci. 12, 783–792. doi: 10.1093/scan/nsw184, PMID: 28008074 PMC5460039

[ref7] Chilver M. R. Park H. R. P. Schofield P. R. Clark C. R. Williams L. M. Gatt J. M. (2022). Emotional face processing correlates with depression/anxiety symptoms but not wellbeing in non-clinical adults: an event-related potential study. J. Psychiatr. Res. 145, 18–26. doi: 10.1016/j.jpsychires.2021.11.038 34844048

[ref8] Damaskinou N. Watling D. (2018). Neurophysiological evidence (ERPs) for hemispheric processing of facial expressions of emotions: evidence from whole face and chimeric face stimuli. Laterality 23, 318–343. doi: 10.1080/1357650X.2017.1361963, PMID: 28857672

[ref9] Delorme A. Makeig S. (2004). EEGLAB: an open source toolbox for analysis of single-trial EEG dynamics including independent component analysis. J. Neurosci. Methods 134, 9–21. doi: 10.1016/j.jneumeth.2003.10.009, PMID: 15102499

[ref10] Diener C. Kuehner C. Brusniak W. Ubl B. Wessa M. Flor H. (2012). A meta-analysis of neurofunctional imaging studies of emotion and cognition in major depression. Neuroimage 61, 677–685. doi: 10.1016/j.neuroimage.2012.04.005, PMID: 22521254

[ref11] Fang F. Potter T. Nguyen T. Zhang Y. C. (2020). Dynamic reorganization of the cortical functional brain network in affective processing and cognitive reappraisal. Int. J. Neural Syst. 30:2050051. doi: 10.1142/S0129065720500513 32812469

[ref12] Fitzgerald P. B. Laird A. R. Maller J. Daskalakis Z. J. (2008). A meta-analytic study of changes in brain activation in depression. Hum. Brain Mapp. 29, 683–695. doi: 10.1002/hbm.20426, PMID: 17598168 PMC2873772

[ref9001] Freeman L. C. (1978). Centrality in social networks: conceptual clarification. Soc. Netw. 1, 215–239.

[ref13] Hagmann P. Cammoun L. Gigandet X. Meuli R. Honey C. J. Wedeen V. J.. (2008). Mapping the structural core of human cerebral cortex. PLoS Biol. 6:e159. doi: 10.1371/journal.pbio.0060159, PMID: 18597554 PMC2443193

[ref14] Hall L. M. J. Klimes-Dougan B. Hunt H. R. Thomas K. M. Houri A. Noack E.. (2014). An fMRI study of emotional face processing in adolescent major depression. J. Affect. Disord. 168, 44–50. doi: 10.1016/j.jad.2014.06.037, PMID: 25036008 PMC4171128

[ref15] Hassan M. Benquet P. Biraben A. Berrou C. Dufor O. Wendling F. (2015). Dynamic reorganization of functional brain networks during picture naming. Cortex 73, 276–288. doi: 10.1016/j.cortex.2015.08.019, PMID: 26478964

[ref16] Henje Blom E. Connolly C. G. Ho T. C. LeWinn K. Z. Mobayed N. Han L.. (2015). Altered insular activation and increased insular functional connectivity during sad and happy face processing in adolescent major depressive disorder. J. Affect. Disord. 178, 215–223. doi: 10.1016/j.jad.2015.03.012, PMID: 25827506 PMC4412607

[ref17] Hillebrand A. Tewarie P. van Dellen E. Yu M. Carbo E. W. S. Douw L.. (2016). Direction of information flow in large-scale resting-state networks is frequency-dependent. Proc. Natl. Acad. Sci. U. S. A. 113, 3867–3872. doi: 10.1073/pnas.1515657113, PMID: 27001844 PMC4833227

[ref18] Ho T. C. Yang G. Wu J. Cassey P. Brown S. D. Hoang N.. (2014). Functional connectivity of negative emotional processing in adolescent depression. J. Affect. Disord. 155, 65–74. doi: 10.1016/j.jad.2013.10.025, PMID: 24268546 PMC4961511

[ref19] Hu B. Tao Y. Yang M. (2023). Detecting depression based on facial cues elicited by emotional stimuli in video. Comput. Biol. Med. 165:107457. doi: 10.1016/j.compbiomed.2023.107457, PMID: 37708718

[ref20] Huang G. Li Y. Zhu H. Feng H. Shen X. Chen Z. (2023). Emotional stimulation processing characteristics in depression: meta-analysis of eye tracking findings. Front. Psychol. 13:1089654. doi: 10.3389/fpsyg.2022.1089654, PMID: 36710847 PMC9880408

[ref21] Jalili M. (2016). Functional brain networks: does the choice of dependency estimator and binarization method matter? Sci. Rep. 6:29780. doi: 10.1038/srep29780, PMID: 27417262 PMC4945914

[ref22] Kaiser R. H. Andrews-Hanna J. R. Wager T. D. Pizzagalli D. A. (2015). Large-scale network dysfunction in major depressive disorder: a meta-analysis of resting-state functional connectivity. JAMA Psychiat. 72, 603–611. doi: 10.1001/jamapsychiatry.2015.0071, PMID: 25785575 PMC4456260

[ref23] Karakaş S. Basar E. (1998). Early gamma response is sensory in origin: a conclusion based on cross-comparison of results from multiple experimental paradigms. Int. J. Psychophysiol. 31, 13–31. doi: 10.1016/s0167-8760(98)00030-0, PMID: 9934618

[ref24] Karamzadeh N. Medvedev A. Azari A. Gandjbakhche A. Najafizadeh L. (2013). Capturing dynamic patterns of task-based functional connectivity with EEG. NeuroImage 66, 311–317. doi: 10.1016/j.neuroimage.2012.10.032, PMID: 23142654 PMC3609939

[ref25] Khanna A. Pascual-Leone A. Michel C. M. Farzan F. (2015). Microstates in resting-state EEG: current status and future directions. Neurosci. Biobehav. Rev. 49, 105–113. doi: 10.1016/j.neubiorev.2014.12.010, PMID: 25526823 PMC4305485

[ref26] Klawohn J. Bruchnak A. Burani K. Meyer A. Lazarov A. Bar-Haim Y.. (2020). Aberrant attentional bias to sad faces in depression and the role of stressful life events: evidence from an eye-tracking paradigm. Behav. Res. Ther. 135:103762. doi: 10.1016/j.brat.2020.103762, PMID: 33160270

[ref27] Kuehl L. K. Deuter C. E. Nowacki J. Ueberrueck L. Wingenfeld K. Otte C. (2021). Attentional bias in individuals with depression and adverse childhood experiences: influence of the noradrenergic system? Psychopharmacology 238, 3519–3531. doi: 10.1007/s00213-021-05969-7, PMID: 34605959 PMC8629860

[ref28] Lai C. Pellicano G. R. Ciacchella C. Guidobaldi L. Altavilla D. Cecchini M.. (2020). Neurophysiological correlates of emotional face perception consciousness. Neuropsychologia 146:107554. doi: 10.1016/j.neuropsychologia.2020.107554, PMID: 32652090

[ref29] Lang P. Bradley M. Cuthbert B. (1999). International affective picture system: technical manual and affective ratings. Gainesville, FL: The Center for Research in Psychophysiology, University of Florida.

[ref30] Lazarov A. Ben-Zion Z. Shamai D. Pine D. S. Bar-Haim Y. (2018). Free viewing of sad and happy faces in depression: a potential target for attention bias modification. J. Affect. Disord. 238, 94–100. doi: 10.1016/j.jad.2018.05.047, PMID: 29870821 PMC6310000

[ref31] Le Van Quyen M. Foucher J. Lachaux J. Rodriguez E. Lutz A. Martinerie J.. (2001). Comparison of hilbert transform and wavelet methods for the analysis of neuronal synchrony. J. Neurosci. Methods 111, 83–98. doi: 10.1016/S0165-0270(01)00372-7, PMID: 11595276

[ref32] Lehmann D. Skrandies W. (1980). Reference-free identification of components of checkerboard-evoked multichannel potential fields. Electroencephalogr. Clin. Neurophysiol. 48, 609–621. doi: 10.1016/0013-4694(80)90419-8, PMID: 6155251

[ref33] Lei L. Zhang Y. Song X. Liu P. Wen Y. Zhang A.. (2021). Face recognition brain functional connectivity in patients with major depression: a brain source localization study by ERP. Front. Psych. 12:662502. doi: 10.3389/fpsyt.2021.662502, PMID: 34803748 PMC8604097

[ref34] Li Y. Cao D. Wei L. Tang Y. Wang J. (2015). Abnormal functional connectivity of EEG gamma band in patients with depression during emotional face processing. Clin. Neurophysiol. 126, 2078–2089. doi: 10.1016/j.clinph.2014.12.026, PMID: 25766267

[ref35] Li Y. Shi W. Liu Z. Li J. Wang Q. Yan X.. (2021). Effective brain state estimation during propofol-induced sedation using advanced EEG microstate spectral analysis. IEEE J. Biomed. Health Inform. 25, 978–987. doi: 10.1109/JBHI.2020.3008052, PMID: 32749987

[ref36] Li X. Q. Wang J. J. (2021). Abnormal neural activities in adults and youths with major depressive disorder during emotional processing: a meta-analysis. Brain Imaging Behav. 15, 1134–1154. doi: 10.1007/s11682-020-00299-2, PMID: 32710330

[ref37] Li M. Zhang J. Jiang C. Wang J. Sun R. Jin S.. (2023). The neural correlates of the recognition of emotional intensity deficits in major depression: an ERP study. Neuropsychiatr. Dis. Treat. 19, 117–131. doi: 10.2147/NDT.S393264, PMID: 36660318 PMC9842523

[ref38] Liu S. Ma R. Luo Y. Liu P. Zhao K. Guo H.. (2021). Facial expression recognition and ReHo analysis in major depressive disorder. Front. Psychol. 12:688376. doi: 10.3389/fpsyg.2021.688376, PMID: 34630204 PMC8493300

[ref39] Liu W. Zhang C. Wang X. Xu J. Chang Y. Ristaniemi T.. (2020). Functional connectivity of major depression disorder using ongoing EEG during music perception. Clin. Neurophysiol. 131, 2413–2422. doi: 10.1016/j.clinph.2020.06.031, PMID: 32828045

[ref40] Lobier M. Siebenhühner F. Palva S. Palva J. M. (2014). Phase transfer entropy: a novel phase-based measure for directed connectivity in networks coupled by oscillatory interactions. Neuroimage 85, 853–872. doi: 10.1016/j.neuroimage.2013.08.056, PMID: 24007803

[ref41] Malhi G. S. Mann J. J. (2018). Depression. Lancet 392, 2299–2312. doi: 10.1016/S0140-6736(18)31948-2 30396512

[ref42] Maslov S. Sneppen K. (2002). Specificity and stability in topology of protein networks. Science 296, 910–913. doi: 10.1126/science.1065103 11988575

[ref43] Mheich A. Hassan M. Khalil M. Berrou C. Wendling F. (2015). A new algorithm for spatiotemporal analysis of brain functional connectivity. J. Neurosci. Methods 242, 77–81. doi: 10.1016/j.jneumeth.2015.01.002 25583381

[ref44] Michel C. M. He B. (2019). EEG source localization. Handb. Clin. Neurol. 160, 85–101. doi: 10.1016/B978-0-444-64032-1.00006-0 31277878

[ref45] Michel C. M. Murray M. M. Lantz G. Gonzalez S. Spinelli L. Grave De Peralta R. (2004). EEG source imaging. Clin. Neurophysiol. 115, 2195–2222. doi: 10.1016/j.clinph.2004.06.001 15351361

[ref46] Monroe S. M. Harkness K. L. (2022). Major depression and its recurrences: life course matters. Annu. Rev. Clin. Psychol. 18, 329–357. doi: 10.1146/annurev-clinpsy-072220-021440, PMID: 35216520

[ref47] Ng B. S. W. Logothetis N. K. Kayser C. (2013). EEG phase patterns reflect the selectivity of neural firing. Cereb. Cortex 23, 389–398. doi: 10.1093/cercor/bhs031, PMID: 22345353

[ref48] Oya H. Kawasaki H. Howard M. R. Adolphs R. (2002). Electrophysiological responses in the human amygdala discriminate emotion categories of complex visual stimuli. J. Neurosci. 22, 9502–9512. doi: 10.1523/JNEUROSCI.22-21-09502.2002, PMID: 12417674 PMC6758059

[ref49] Pan D. Hoid D. Gu R. Li X. (2020). Emotional working memory training reduces rumination and alters the EEG microstate in anxious individuals. Neuroimage Clin. 28:102488. doi: 10.1016/j.nicl.2020.102488, PMID: 33395979 PMC7689328

[ref50] Price J. L. Drevets W. C. (2012). Neural circuits underlying the pathophysiology of mood disorders. Trends Cogn. Sci. 16, 61–71. doi: 10.1016/j.tics.2011.12.011 22197477

[ref51] Rizkallah J. Benquet P. Kabbara A. Dufor O. Wendling F. Hassan M. (2018). Dynamic reshaping of functional brain networks during visual object recognition. J. Neural Eng. 15:056022. doi: 10.1088/1741-2552/aad7b1, PMID: 30070974

[ref52] Rossion B. (2014). Understanding face perception by means of human electrophysiology. Trends Cogn. Sci. 18, 310–318. doi: 10.1016/j.tics.2014.02.013, PMID: 24703600

[ref53] Rubinov M. Sporns O. (2010). Complex network measures of brain connectivity: uses and interpretations. NeuroImage 52, 1059–1069. doi: 10.1016/j.neuroimage.2009.10.003, PMID: 19819337

[ref54] Schendan H. E. Ganis G. Kutas M. (1998). Neurophysiological evidence for visual perceptual categorization of words and faces within 150 ms. Psychophysiology 35, 240–251. doi: 10.1111/1469-8986.3530240, PMID: 9564744

[ref55] Schindler S. Bruchmann M. Gathmann B. Moeck R. Straube T. (2021). Effects of low-level visual information and perceptual load on P1 and N170 responses to emotional expressions. Cortex 136, 14–27. doi: 10.1016/j.cortex.2020.12.011, PMID: 33450599

[ref56] Schyns P. G. Thut G. Gross J. (2011). Cracking the code of oscillatory activity. PLoS Biol. 9:e1001064. doi: 10.1371/journal.pbio.1001064, PMID: 21610856 PMC3096604

[ref57] Sereno M. I. Huang R. (2006). A human parietal face area contains aligned head-centered visual and tactile maps. Nat. Neurosci. 9, 1337–1343. doi: 10.1038/nn1777, PMID: 16998482

[ref58] Shim M. Im C. Kim Y. Lee S. (2018). Altered cortical functional network in major depressive disorder: a resting-state electroencephalogram study. Neuroimage Clin. 19, 1000–1007. doi: 10.1016/j.nicl.2018.06.012, PMID: 30003037 PMC6039896

[ref59] Slonim D. A. Yehezkel I. Paz A. Bar-Kalifa E. Wolff M. Dar A.. (2023). Facing change: using automated facial expression analysis to examine emotional flexibility in the treatment of depression. Admin. Pol. Ment. Health. doi: 10.1007/s10488-023-01310-w, [Online ahead of print] 37880472

[ref60] Smith K. (2014). Mental health: a world of depression. Nature 515, 180–181. doi: 10.1038/515180a 25391942

[ref61] Song J. Davey C. Poulsen C. Luu P. Turovets S. Anderson E.. (2015). EEG source localization: sensor density and head surface coverage. J. Neurosci. Methods 256, 9–21. doi: 10.1016/j.jneumeth.2015.08.015, PMID: 26300183

[ref62] Tian L. Wang J. Yan C. He Y. (2011). Hemisphere- and gender-related differences in small-world brain networks: a resting-state functional MRI study. Neuroimage 54, 191–202. doi: 10.1016/j.neuroimage.2010.07.066, PMID: 20688177

[ref63] Tong Y. Zhao G. Zhao J. Xie N. Han D. Yang B.. (2020). Biases of happy faces in face classification processing of depression in Chinese patients. Neural Plast. 2020, 1–8. doi: 10.1155/2020/7235734, PMID: 32879624 PMC7448107

[ref64] von Wegner F. Bauer S. Rosenow F. Triesch J. Laufs H. (2021). EEG microstate periodicity explained by rotating phase patterns of resting-state alpha oscillations. NeuroImage 224:117372. doi: 10.1016/j.neuroimage.2020.117372, PMID: 32979526

[ref65] Wang J. Wei Q. Yuan X. Jiang X. Xu J. Zhou X.. (2018). Local functional connectivity density is closely associated with the response of electroconvulsive therapy in major depressive disorder. J. Affect. Disord. 225, 658–664. doi: 10.1016/j.jad.2017.09.001, PMID: 28910748

[ref66] Wang C. Xu J. Zhao S. Lou W. (2016). Graph theoretical analysis of EEG effective connectivity in vascular dementia patients during a visual oddball task. Clin. Neurophysiol. 127, 324–334. doi: 10.1016/j.clinph.2015.04.063, PMID: 26093934

[ref67] Wang S. Zhang D. Fang B. Liu X. Yan G. Sui G.. (2021). A study on resting EEG effective connectivity difference before and after neurofeedback for children with ADHD. Neuroscience 457, 103–113. doi: 10.1016/j.neuroscience.2020.12.038, PMID: 33476697

[ref68] Watts D. J. Strogatz S. H. (1988). Collective dynamics of ‘small-world’ networks. Nature 393, 440–442. doi: 10.1038/30918 9623998

[ref69] Whittle S. Allen N. B. Lubman D. I. Yücel M. (2006). The neurobiological basis of temperament: towards a better understanding of psychopathology. Neurosci. Biobehav. Rev. 30, 511–525. doi: 10.1016/j.neubiorev.2005.09.003, PMID: 16289282

[ref70] Whitton A. E. Deccy S. Ironside M. L. Kumar P. Beltzer M. Pizzagalli D. A. (2017). Electroencephalography source functional connectivity reveals abnormal high-frequency communication among large-scale functional networks in depression. Biol. Psychiatry Cogn. Neurosci. Neuroimaging 3, 50–58. doi: 10.1016/j.bpsc.2017.07.001, PMID: 29397079 PMC5801763

[ref71] Willinger D. Karipidis I. I. Haberling I. Berger G. Walitza S. Brem S. (2022). Deficient prefrontal-amygdalar connectivity underlies inefficient face processing in adolescent major depressive disorder. Transl. Psychiatry 12:195. doi: 10.1038/s41398-022-01955-5, PMID: 35538052 PMC9090758

[ref72] Xia M. Wang J. He Y. (2013). BrainNet viewer: a network visualization tool for human brain connectomics. PLoS One 8:e68910. doi: 10.1371/journal.pone.0068910, PMID: 23861951 PMC3701683

[ref73] Yoshimura M. Pascual-Marqui R. D. Nishida K. Kitaura Y. Mii H. Saito Y.. (2019). Hyperactivation of the frontal control network revealed by symptom provocation in obsessive-compulsive disorder using EEG microstate and sLORETA analyses. Neuropsychobiology 77, 176–185. doi: 10.1159/000491719, PMID: 30248667

[ref74] Zhang D. He Z. Chen Y. Wei Z. (2016). Deficits of unconscious emotional processing in patients with major depression: an ERP study. J. Affect. Disord. 199, 13–20. doi: 10.1016/j.jad.2016.03.056, PMID: 27057648

[ref75] Zhang M. H. Zhou H. Y. Liu L. Q. Feng L. Yang J. Wang G.. (2018). Randomized EEG functional brain networks in major depressive disorders with greater resilience and lower rich-club coefficient. Clin. Neurophysiol. 129, 743–758. doi: 10.1016/j.clinph.2018.01.017, PMID: 29453169

[ref76] Zhao Z. Y. Niu Y. X. Zhao X. F. Zhu Y. Shao Z. P. Wu X. Y.. (2022). EEG microstate in first-episode drug-naive adolescents with depression. J. Neural Eng. 19:056016. doi: 10.1088/1741-2552/ac88f6, PMID: 35952647

[ref77] Zhao Q. Tang Y. Chen S. Lyu Y. Curtin A. Wang J.. (2015). Early perceptual anomaly of negative facial expression in depression: an event-related potential study. Neurophysiol. Clin. 45, 435–443. doi: 10.1016/j.neucli.2015.09.011 26602972

