# Supplementary material for: Disrupted properties of functional brain networks in major depressive disorder during emotional face recognition: an EEG study via graph theory analysis
Source: Front Hum Neurosci. 2024 Feb 13;18:1338765. doi: 10.3389/fnhum.2024.1338765 (PMC10897049; doi:10.3389/fnhum.2024.1338765)
Supplement: Supplementary file 1 [file Data_Sheet_1.docx]

Supplementary Material

Abbreviations: MDD, major depressive disorder; HC, healthy controls

**
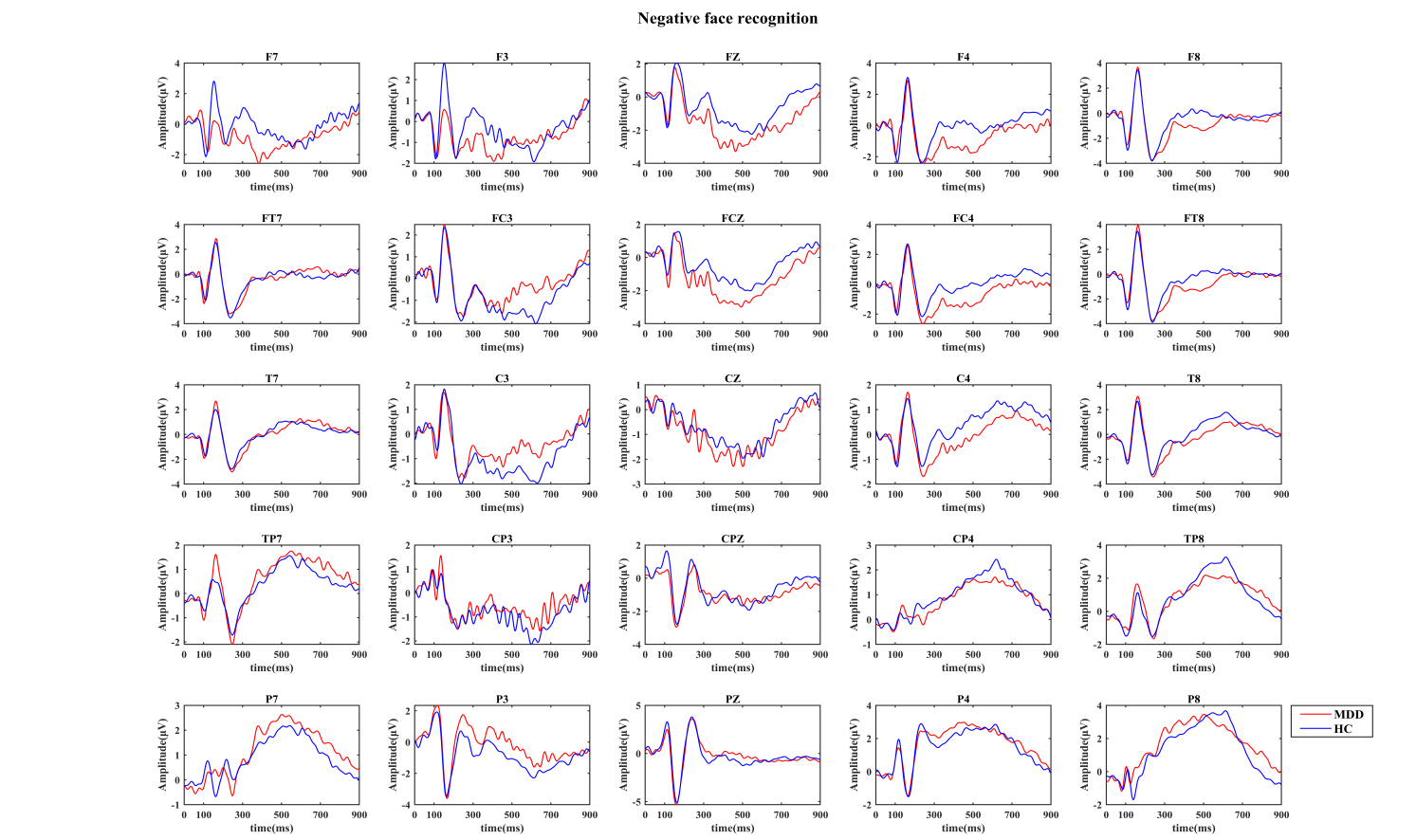
**

**FIGURE 1S | The visual comparison of event-related potential (ERP) signals between MDD and HC groups for individual channel under negative face recognition.** **It was found that the ERP waves of two groups were different in multiple brain areas.**


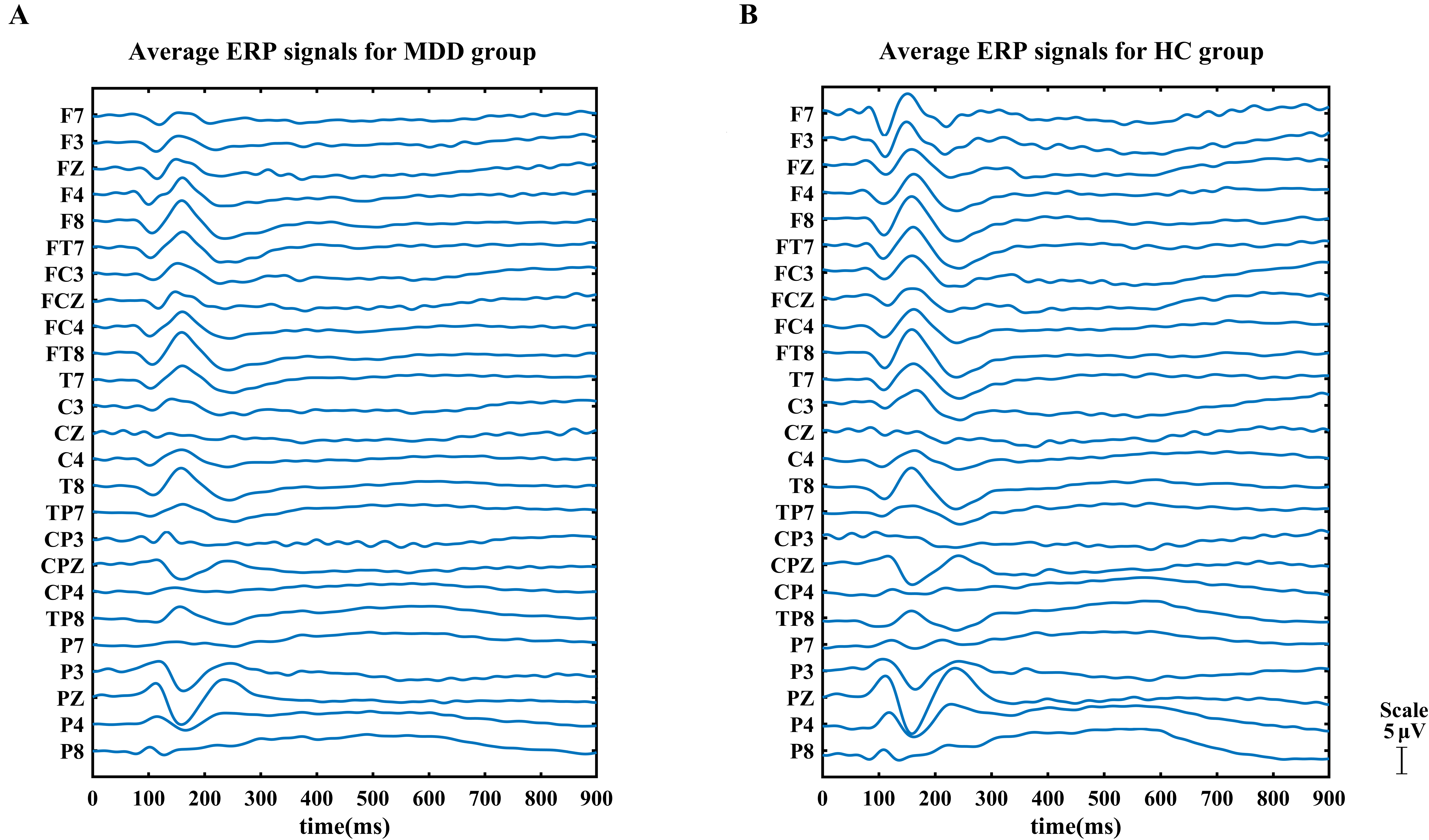


**FIGURE 2S | Event-related potential (ERP) signals for positive face recognition task: (A) MDD group, (B) HC group. We can observed that the ERP signals of two groups were different, especially in the frontal area.**

**
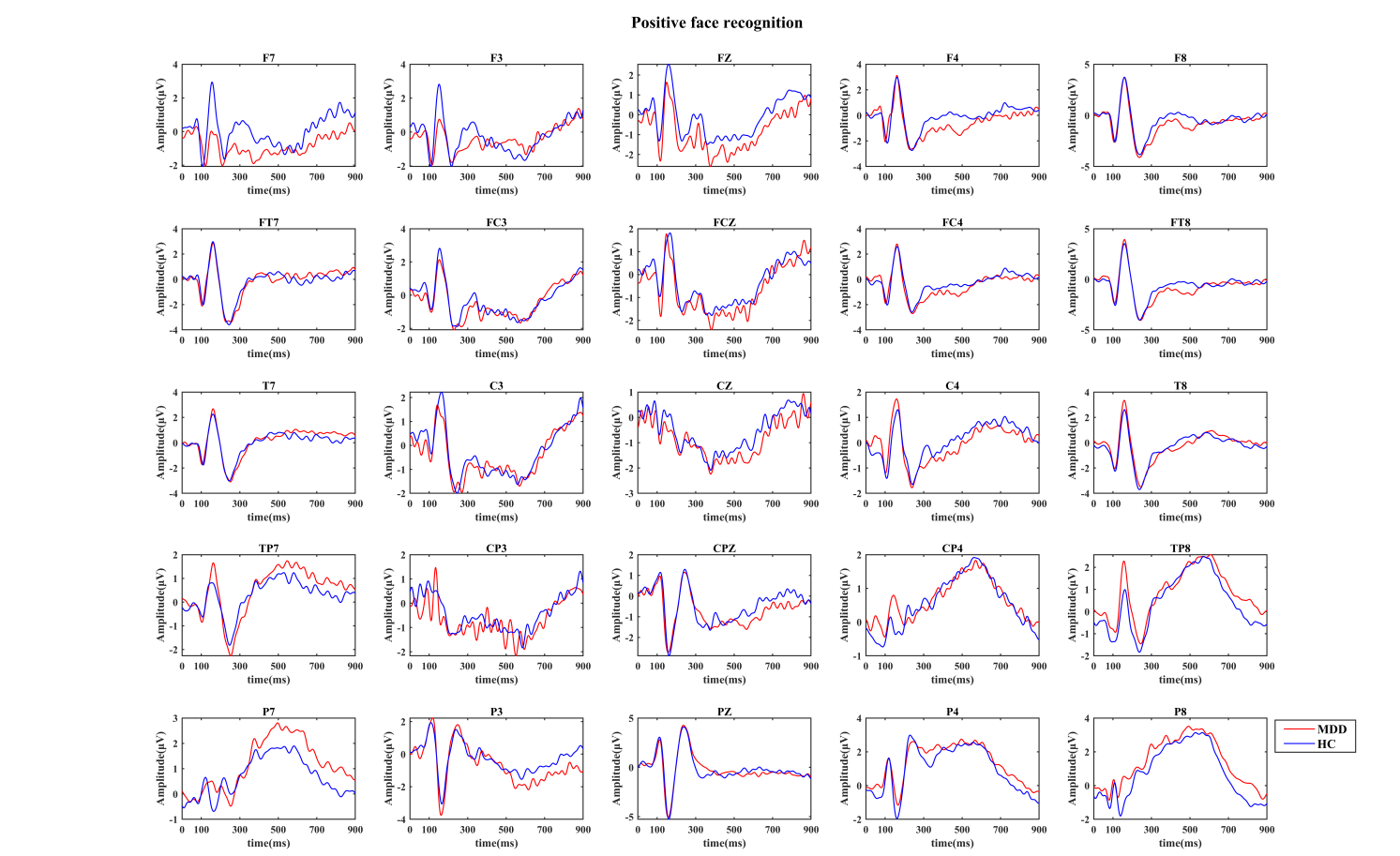
**

**FIGURE 3S | The visual comparison of event-related potential (ERP) signals between MDD and HC groups for individual channel under positive face recognition. It was found that the ERP waves of two groups were different in multiple brain areas.**


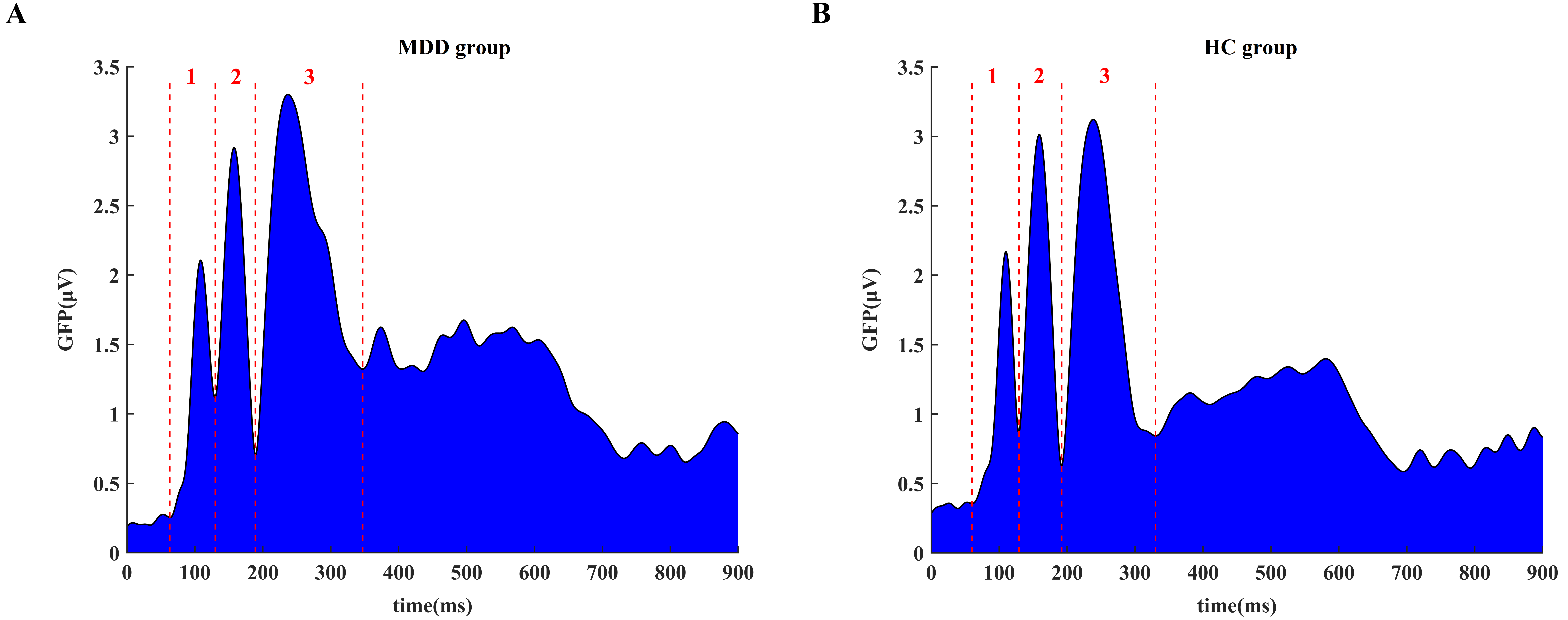


**Figure 4S.** Global field potential (GFP) of group-averaged event-related potential (ERP) signals for positive face recognition task: (A) MDD group, (B) HC group. The process was divided into several stages for both two groups according to the local minima of GFP.

**Table S1****.** Significantly statistical results of normalized clustering coefficient between the MDD and HC groups for multiple threshold values at the first stage of the negative faces processing.

| Threshold | Statistic | | Effect sizes | MDD group (Mean ± SD) | HC group (Mean ± SD) | 95% confidence interval for difference | | *p* |
| --- | --- | --- | --- | --- | --- | --- | --- | --- |
|  | t | U |  |  |  | Lower limit | Upper limit |  |
| 0.320 | － | 84 | -3.433 | 1.0026 ± 0.0031 | 1.0049 ± 0.0043 | － | － | 0.039 |
| 0.330 | － | 81 | -3.818 | 1.0024 ± 0.0026 | 1.0048 ± 0.0043 | － | － | 0.030 |
| 0.340 | － | 86 | -2.625 | 1.0026 ± 0.0031 | 1.0044 ± 0.0045 | － | － | 0.046 |
| 0.355 | -2.208 | － | -4.291 | 1.0021 ± 0.0021 | 1.0041 ± 0.0031 | -0.0039 | -0.0002 | 0.035 |
| 0.380 | － | 84 | -4.375 | 1.0020 ± 0.0018 | 1.0039 ± 0.0030 | － | － | 0.039 |
| 0.385 | － | 76 | -4.226 | 1.0019 ± 0.0020 | 1.0039 ± 0.0032 | － | － | 0.018 |
| 0.390 | － | 78 | -4.360 | 1.0021 ± 0.0017 | 1.0037 ± 0.0024 | － | － | 0.022 |
| 0.400 | -2.353 | － | -4.573 | 1.0021 ± 0.0017 | 1.0038 ± 0.0025 | -0.0033 | -0.0002 | 0.025 |
| 0.415 | -2.135 | － | -4.150 | 1.0019 ± 0.0017 | 1.0034 ± 0.0024 | -0.0030 | -0.0001 | 0.040 |
| 0.420 | -2.083 | － | -4.049 | 1.0020 ± 0.0017 | 1.0033 ± 0.0021 | -0.0027 | 0.0000 | 0.045 |
| 0.435 | -2.444 | － | -4.593 | 1.0019 ± 0.0013 | 1.0035 ± 0.0024 | -0.0029 | -0.0003 | 0.021 |
| 0.440 | -2.082 | － | -3.927 | 1.0020 ± 0.0016 | 1.0035 ± 0.0027 | -0.0031 | 0.0000 | 0.047 |

“－” denotes none.

**Table S2.** Significantly statistical results of normalized characteristic path length between the MDD and HC groups for multiple threshold values at the third stage of the positive faces processing.

| Threshold | Statistic | | Effect sizes | MDD group (Mean ± SD) | HC group (Mean ± SD) | 95% confidence interval for difference | | *p* |
| --- | --- | --- | --- | --- | --- | --- | --- | --- |
|  | t | U |  |  |  | Lower limit | Upper limit |  |
| 0.340 | － | 83 | -2.883 | 0.9701 ± 0.0324 | 0.9853 ± 0.0273 | － | － | 0.036 |
| 0.345 | － | 82 | -3.469 | 0.9679 ± 0.0358 | 0.9875 ± 0.0283 | － | － | 0.033 |
| 0.355 | － | 80.5 | -4.439 | 0.9577 ± 0.0493 | 0.9867 ± 0.0207 | － | － | 0.027 |
| 0.365 | － | 64 | -4.912 | 0.9539 ± 0.0502 | 0.9892 ± 0.0296 | － | － | 0.005 |
| 0.370 | － | 68.5 | -5.287 | 0.9462 ± 0.0615 | 0.9883 ± 0.0219 | － | － | 0.008 |
| 0.375 | － | 56 | -6.122 | 0.9473 ± 0.0567 | 0.9931 ± 0.0230 | － | － | 0.002 |
| 0.380 | － | 54 | -7.306 | 0.9389 ± 0.0523 | 0.9903 ± 0.0241 | － | － | 0.001 |
| 0.385 | － | 66 | -6.136 | 0.9402 ± 0.0569 | 0.9878 ± 0.0280 | － | － | 0.006 |
| 0.390 | -3.015 | － | -6.148 | 0.9220 ± 0.0768 | 0.9828 ± 0.0264 | -0.1032 | -0.0185 | 0.007 |
| 0.395 | -3.392 | － | -6.862 | 0.9353 ± 0.0542 | 0.9853 ± 0.0246 | -0.0808 | -0.0193 | 0.003 |
| 0.400 | -3.586 | － | -7.210 | 0.9332 ± 0.0582 | 0.9916 ± 0.0310 | -0.0922 | -0.0247 | 0.002 |
| 0.405 | － | 83 | -3.909 | 0.9563 ± 0.0577 | 0.9885 ± 0.0341 | － | － | 0.036 |
| 0.410 | -2.226 | － | -4.328 | 0.9483 ± 0.0557 | 0.9843 ± 0.0377 | -0.0689 | -0.0031 | 0.033 |

“－” denotes none.
